# A modified weighted chimp optimization algorithm for training feed-forward neural network

**DOI:** 10.1371/journal.pone.0282514

**Published:** 2023-03-28

**Authors:** Eman A. Atta, Ahmed F. Ali, Ahmed A. Elshamy

**Affiliations:** 1 Department of Mathematics, Faculty of Science, Suez Canal University, Ismailia, Egypt; 2 Faculty of Computers and Informatics, Suez Canal University, Ismailia, Egypt; Vellore Institute of Technology: VIT University, INDIA

## Abstract

Swarm intelligence algorithms (SI) have an excellent ability to search for the optimal solution and they are applying two mechanisms during the search. The first mechanism is exploration, to explore a vast area in the search space, and when they found a promising area they switch from the exploration to the exploitation mechanism. A good SI algorithm can balance the exploration and the exploitation mechanism. In this paper, we propose a modified version of the chimp optimization algorithm (ChOA) to train a feed-forward neural network (FNN). The proposed algorithm is called a modified weighted chimp optimization algorithm (MWChOA). The main drawback of the standard ChOA and the weighted chimp optimization algorithm (WChOA) is they can be trapped in local optima because most of the solutions update their positions based on the position of the four leader solutions in the population. In the proposed algorithm, we reduced the number of leader solutions from four to three, and we found that reducing the number of leader solutions enhances the search and increases the exploration phase in the proposed algorithm, and avoids trapping in local optima. We test the proposed algorithm on the Eleven dataset and compare it against 16 SI algorithms. The results show that the proposed algorithm can achieve success to train the FNN when compare to the other SI algorithms.

## 1 Introduction

Using machine learning approaches, classification [1, 2], function approximation, patter recognition [3], prediction [4] and others [5, 6] have become common applications in a variety of academic subjects [7]. Groundwater management problems, data mining, climatic and environmental problems, pharmaceuticals, engineering design issues, image segmentation, power flow, solar PV modules, and other topics are considered to be the most well-known applications that used neural networks to solve [8–10]. Artificial neural networks (ANN) are undoubtedly ranked among the most reputable methods in this field, that have been extensively used to solve various issues. ANN [11–13] is inspired by non-parametric mathematical models of physiological neural networks [14]. In the subject of Computational Intelligence, ANN are one of the most important inventions. They typically handle classification problems by stimulating neurons in the human brain [15–17]. On 1943, the first primitive conceptions of NNs were developed [18]. Feed-forward network [19], Radial basis function (RBF) network [20], Recurrent neural network [21], and convolutional neural network(CNN) [22]. The FNN is the most popular among them due to its straightforward design and effective functionality [23–25]. ANN have a high level of performance and simple to implement, and they can capture the hidden relationship between the inputs. Furthermore, ANNs can be implemented in parallel architectures and have excellent scalability, thus they can benefit from current technological breakthroughs in this situation [26, 27]. ANNs have a remarkable ability to tackle difficult problems such as function approximation [28], data classification [29], image recognition [30], control of nonlinear systems modelling [31], and environmental forecasting [32]. The ability to learn is one of the most important qualities of an ANN. ANN can be changed by modifying its structure. There are four main learning procedures for neural networks: supervised learning [33], unsupervised learning [34], reinforcement learning [35], and meta-heuristic learning [36].

When the problem outputs are known in advance, such as in pattern recognition and classification tasks, supervised learning is utilised. The back-propagation (BP) method [37], which is a gradient-based technique, is a typical supervised learning strategy used in ANN. Slow convergence and premature convergence to local optimum are two shortcomings of BP that make it unsuitable for practical applications [38, 39].

When the outputs are missing or uncertain, unsupervised learning is used. Text categorization and clustering applications typically use unsupervised learning [40]. Reinforcement learning on the other hand is utilised when the problem has a complex stochastic structure and is difficult to evaluate, such as control optimization problems. Meta-heuristic algorithms [41, 42] are search strategies for locating a sufficiently good solution to optimization issues. Meta-heuristic learning can estimate optimal or semi-optimal connection weights for ANN with a lower chance of getting stuck in the many local optima in the search space [43]. ANN have been trained using a variety of meta-heuristic learning algorithms [44], including the Genetic Algorithm (GA) [45], Particle Swarm Optimization (PSO) [46], Evolutionary Strategies (ES) [47], Ant Colony Optimization (ACO) [48], Cuckoo Search (CS) [49], Firefly Algorithm (FA) [50], Population-Based Incremental Learning (PBIL) [51], Differential Evolution (DE) [52], Artificial Bee Colony (ABC) [53], and many other algorithms. The well-known No Free Lunch theorem (NFL) [54–56] has demonstrated that no superior meta-heuristic algorithm exists can perfectly learn ANN and handle all types of issues. Local optimization is probably reduced by GA although it converges more slowly. In applications that call on real-time processing, it performs poorly. ABC requires complex computations. The ES algorithm performs poorly since it was built using different mutation techniques. Evolutionary algorithm use of mutation preserves population diversity and encourages exploitation, which is one of the primary causes of ES subpar performance. Additionally, this programme uses a deterministic method for selecting individuals. As a result, choosing a person is less random, and local optima avoidance is also less random. GWO fall into the trap of local optimization despite their low complexity and quick convergence, hence they are unsuitable for issues involving local optimization. The SSA algorithm’s intricacy and abundance of regulatory parameters are two of its flaws. DE is unsuitable for real-time use due to its numerous control settings and time-consuming calculations. Actually, the primary driving force behind this work is the fact that existing multi-solution stochastic trainers are still susceptible to local optima stagnation. As a result of all of these factors, many academics are turning to other meta-heuristic approaches to train ANN. The imbalance between the two phases of exploration and extraction is the primary cause of local optimizations getting stuck. In order to address the two main problems of slow convergence and trapping in local optima when solving optimization problems, this paper proposes a Modified Weighted ChOA (MWChOA) for training a multi-layer feed-forward neural network model. The solutions in the proposed algorithm update thier positions based on the position of the three leader solutions instead of the four leader solutions in the standard ChOA algorithm. The applied modification in the proposed algorithm achieves the balance between exploration and exploitation and can help it avoids trapping in local optima.

The main contribution of this paper is as following:

1. A new modified version of the standard ChOA and the WChOA are introduced to find the optimal weights and bias in the FNN.
2. We reduced the number of the leader solutions from Four to three to balance between the exploration and the exploitation processes and avoid stuck in local optima.
3. The proposed algorithm is tested on the Eleven benchmark dataset and compared against 16 SI algorithms.

The remaining sections of the paper are arranged as follows: Section 2 of the paper introduces several relevant publications in the recent work. The description and structure of a multilayer feedforward neural network (FNN) are introduced in Section 3. The Proposed Algorithm MWChOA is introduced in Section 4. The experimental result are reported in Section 5. Section 6 summarises the content of this work and offers some suggestions for future research.

## 2 Related work

In the recent decade, ANN learning has gotten a lot of attention as a way to increase the efficiency of ANN modelling outputs. Many researchers have successfully trained neural networks using various well-known metaheuristic optimization techniques. In this section, we will present a review of some studies using metaheuristic optimization techniques for training neural networks.

One of the first meta-heuristic algorithm for training feed-forward neural networks was the Genetic Algorithm (GA) [57]. Some researchers have used enhanced GA to train neural networks [58]. The weights and network topology of the MLP networks were evolved using particle swarm optimization (PSO) in [59]. Based on, the educational approach was modified

PSO outperformed other optimizers in terms of accuracy. Other researchers have used modified PSO in studies like [60]. In [46], a hybrid approach combining a PSO optimizer to understand MLP, back-propagation was suggested. The technique was evaluated utilising various data classification issues and used to the learning of MLP networks. PSO algorithm was used to train feed-forward neural networks by Ismail and other researchers in 2005 [61].

To address the continuous optimization, the authors presented the Ant Colony Optimization algorithm (ACO) in [62]. The ACO was also integrated with other gradient-based techniques, including Levenberg-Marquardt and back-propagation algorithms. Socha et al. trained a feed-forward neural network using the ant colony optimization (ACO) algorithm in 2007 [63]. Karaboga and other researchers employed artificial bee colonies (ABC) and enhanced ABC algorithms to train feed-forward neural networks between 2007 and 2011 [64]. In 2019, Ghorbani et al. [65] trained feed-forward neural networks using an improved gravitational search algorithm (GSA). In 2011, Mirjalili et al. utilised the magnetic optimization algorithm (MOA) to train feed-forward neural networks, and in 2015, they employed the grey wolf optimizer (GWO) to train them [66, 67]. To train a feed-forward neural network, Pu et al. employed a novel hybrid biogeography-based optimization technique in 2018 [68]. To train a feed-forward neural network, Zhao et al. used a selfish herds optimization technique with orthogonal architecture and information updating in 2019 [69]. To train a feed-forward neural network, Xu J et al. employed a hybrid nelder–mead and dragonfly algorithm in 2019 [70]. In their paper [71], Goerick et al. proposed a novel MLP learning mechanism based on the Evolution Strategy (ES). Ilonen et al. used the differential evolution (DE) optimization method to improve the MLP learning process in [52]. Aljarah, et al. trained neural network using the bird swarm algorithm (BSA) algorithm in 2019 [72]. In 2021 Sağ et al.used vortex search optimization algorithm for training feed-forward neural network [73].In 2022 Chatterjee et al. proposed a Chaotic oppositional-based whale optimization to train a feed forward neural network [74]. In 2022 Gülcü [75] trained feed-forward neural networks using dragonfly algorithm. In 2023 KUMAR [76] use ZEALOUS-PSO to train multilayer perceptron neural networks. Emambocus in 2023 made a Survey on training neural network using different types of optimization algorithms [77]. And others researchers have recently been using recent-swarm intelligence algorithms for training feedforward neural networks [78–84].

## 3 Feed forward neural network (FNN)

**Fig 1:**
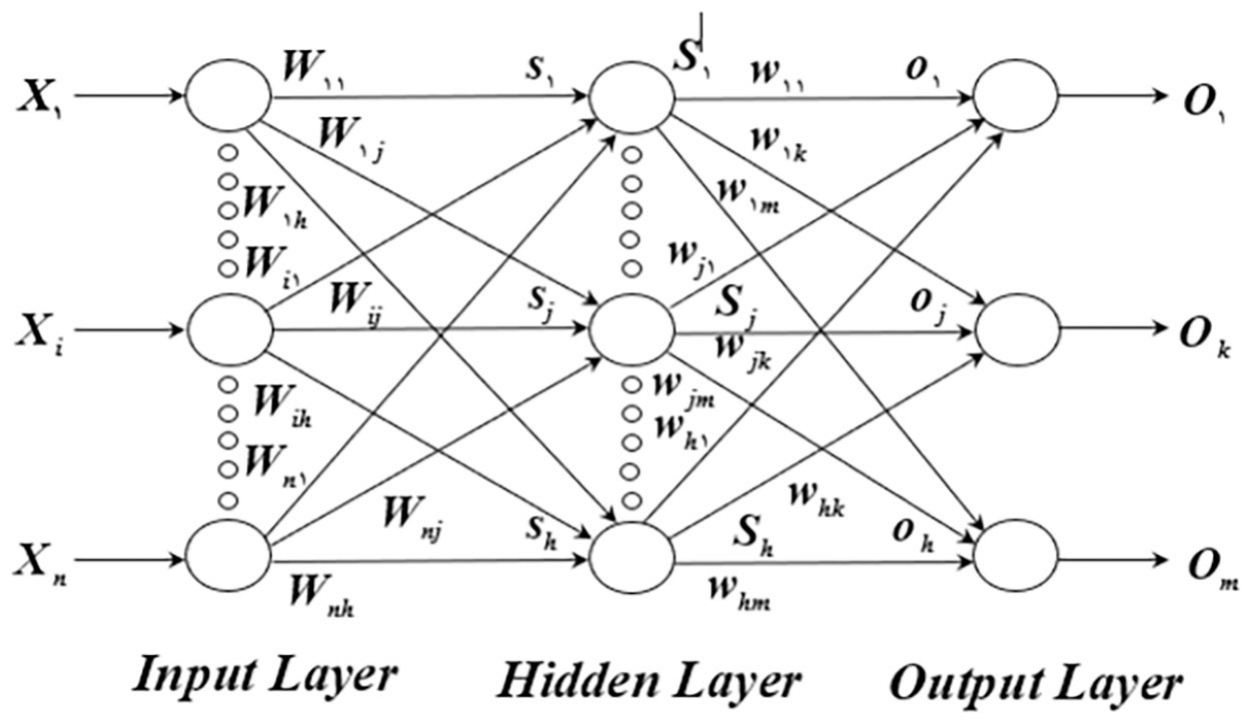
The structure of the FNN.

The multilayer feed-forward neural network structure is composed of three layers: an input layer, a hidden layer, and an output layer. The hidden layer may consist of one or more layers, and neurons are arranged parallel to one another in each layer [85]. A feed-forward neural network (FNN) with three layers is seen in Fig 1. There is just one hidden layer, and there are *h* hidden layer nodes in total. The output layer has *m* nodes. The input layer contains *n* input nodes. Weight [86] is the one-way connection of nodes between adjacent layers. The FNN depicted in Fig 1 is one in which the hidden layer receives n data from the input layer after which they are multiplied by their corresponding weights. As the neurons go through the hidden layer, the sigmoid function processes them as the hidden layer’s output value and multiplies them with the appropriate weights as input to the output layer. The sigmoid transfer function and output are used to calculate the output layer.

First, the total weighted of the input layer is calculated using Eq 1:

$$\begin{array}{c} s_{j}=\sum_{i=1}^{n}W_{ij}X_{i}-\theta_{j},\;\;\;j=1,2,\ldots .h \end{array}$$ (1)

Where *n* is the number of input nodes, *W*_*ij*_, displays the connection weight from node *i*^*th*^ in the input layer to *j*^*th*^ node in the hidden layer *h*, *θ*_*j*_ is the bias of the hidden node *j*^*th*^, and *X*_*i*_ denotes the input of *i*^*th*^. The deviation (threshold value) of the first hidden layer and the first input is among them and is represented by the node from the first node to the first hidden layer of the input layer. The output value of the hidden layer node is then determined by Eq 2: using the sigmoid function.

$$\begin{array}{c} S_{j}=sigmoid\left(s_{j}\right)=\frac{1}{\text{exp}(-\left(s_{j}\right))},\;\;\;j=1,2,\ldots .h \end{array}$$ (2)

Using Eqs 3 and 4 to input values to the output layer in the same way as the hidden layer’s output:

$$\begin{array}{c} o_{k}=\sum_{i=1}^{n}W_{ij}S_{j}-\theta_{k}^{\prime },\;\;\;k=1,2,\ldots .m \end{array}$$ (3)

$$\begin{array}{c} O_{k}=sigmoid\left(o_{k}\right)=\frac{1}{\text{exp}(-\left(o_{k}\right))},\;\;\;k=1,2,\ldots .m \end{array}$$ (4)

*W*_*jk*_ is the connection weight from the *j*^*th*^ hidden node to the *k*^*th*^ output node, and *k* is the *k*^*th*^ output node’s bias (threshold). The relation weights and bias values are the most critical aspects of the FNN, as shown in Eqs 1, 2, 3 and 4 and they define the final output value. The main aim of FNN training is to find the best weights and bias values for a given input and achieve the most ideal output.

## 4 The proposed MWChOA algorithm

We describe the main structure of the proposed MWChOA algorithm in the following subsections.

### 4.1 Chimp optimization algorithm (ChOA)

In the following subsections, we highlight the social life, inspiration, and structure of the standard ChOA as follows.

#### 4.1.1 Social life and inspiration

The Chimps are a kind of ape and they are considered one of the most intelligent animals in the world due to their big brain relative to their body ratio. They are living in groups. Each group tries to discover the environment (search space) with different strategies. In a chimp group, each individual has a different method of hunting. There are four types of individuals that are responsible for the hunting in the group. These individuals are called drivers, barriers, chasers, and attackers. The drivers are responsible for pursuing the prey without catching it. The barriers are constructing a dam to avoid the progression of the prey. The chasers pursue the prey rapidly to catch up with it. In order to make the prey return to the location of the chasers, the attackers finally foresaw the getaway route. The other individuals in the group follow the four leaders (drivers, barriers, chasers, and attackers) to hunt the prey by updating their position based on the leader’s position. The chimps have a distinct social behavior in the final stage of hunting which is a sexual motivation by leaving their hunting duties and trying to search for food randomly.

#### 4.1.2 Chimp optimization algorithm implementation

The Chimp Optimization Algorithm (ChOA) is a recent natural inspired algorithm, which simulates the chimp’s life and their behavior in the hunting process. The ChOA is proposed in 2020 by M.Khishe et al [87]. In this subsection, we simulate the social behavior of the chimps by specifying the mathematical model of the chimp optimization algorithm (ChOA) as follows. The population in the ChOA is different than the other swarm intelligence algorithms. It contains four groups which are drivers *D*, barriers *B*, chasers *C*, and attackers *A*. The pray represents the optimal solution, however, it is hard to know its location in the search space. During the search, The four leaders are assigned as follows.

- **The attacker individual** $\vec{X_{A}}$ . The attackers’ group represents the exploitation process in the ChAO. The individual $\vec{X_{1}}$ represents the first solution in the group, while the $\vec{D_{A}}$ represents the distance between the position of the current solution $\vec{X}$ and the position of the attacker solution $\vec{X_{A}}$ as shown in the following equations.
  $$\begin{array}{c} \vec{D_{A}}=\left|\vec{C_{1}}\vec{X_{A}}-\vec{M_{1}}\vec{X}\right|,\\ \vec{X_{1}}=\left|\vec{X_{A}}-\vec{A_{1}}\vec{D_{A}}\right| \end{array}$$ (5)
- **The barrier individual** $\vec{X_{B}}$ . The barrier group participates with the chaser and the barrier groups in the exploitation process in the ChOA. The individual $\vec{X_{2}}$ represents the second solution in the group, while the $\vec{D_{B}}$ represents the distance between the position of the current solution $\vec{X}$ and the position of the barrier solution $\vec{X_{B}}$ as shown in the following equations.
  $$\begin{array}{c} \vec{D_{B}}=\left|\vec{C_{2}}\vec{X_{B}}-\vec{M_{2}}\vec{X}\right|,\\ \vec{X_{2}}=\left|\vec{X_{B}}-\vec{A_{2}}\vec{D_{B}}\right| \end{array}$$ (6)
- **The chaser individual** $\vec{X_{C}}$ . The chaser group is part of the exploitation process in the ChOA. The individual $\vec{X_{3}}$ represents the third solution in the group, while the $\vec{D_{C}}$ represents the distance between the position of the current solution $\vec{X}$ and the position of the chaser solution $\vec{X_{C}}$ as shown in the following equations.
  $$\begin{array}{c} \vec{D_{C}}=\left|\vec{C_{3}}\vec{X_{C}}-\vec{M_{3}}\vec{X}\right|,\\ \vec{X_{3}}=\left|\vec{X_{C}}-\vec{A_{3}}\vec{D_{C}}\right| \end{array}$$ (7)
- **The driver individual** $\vec{X_{D}}$ . The driver group is a stage in the exploitation process in the ChOA. The individual $\vec{X_{4}}$ represents the fourth solution in the group, while the $\vec{D_{D}}$ represents the distance between the position of the current solution $\vec{X}$ and the position of the driver solution $\vec{X_{D}}$ as shown in the following equations.
  $$\begin{array}{c} \vec{D_{D}}=\left|\vec{C_{4}}\vec{X_{D}}-\vec{M_{4}}\vec{X}\right|,\\ \vec{X_{4}}=\left|\vec{X_{D}}-\vec{A_{4}}\vec{D_{D}}\right| \end{array}$$ (8)

The vectors $\vec{A},\vec{C}$, and $\vec{M}$ have a great effect on the algorithm performance and their values are calculated as shown in the following subsection.

#### 4.1.3 The main paraments of the algorithm

The ChOA has three main parameters, these parameters are the vectors $\vec{A}$, $\vec{C}$, and $\vec{M}$, and they are calculated as follows. The vector $\vec{A}$ is responsible for switching from the exploration to the exploitation phases. The value of $\vec{A}$ lies in the range [−2*f*, 2*f*].

$$\begin{array}{c} \vec{A}=2\cdot f\cdot \vec{r_{1}}-f \end{array}$$ (9)

The vector $\vec{C}$ is a random vector in the range [0, 2]. It is applied in the ChoA to increase the diversity of the algorithm and help it to escape from local optima.

$$\begin{array}{c} \vec{C}=2\cdot \vec{r_{2}} \end{array}$$ (10)

The vector $\vec{M}$ represents the sexual motivation in the ChOA and it is computed based on the chaotic map.

$$\begin{array}{c} \vec{M}=\text{Chaotic}\;\text{value} \end{array}$$ (11)

Where the vectors $\vec{r_{1}}$ and $\vec{r_{2}}$ are random vectors in [0, 1]. *f* is a control value that is reduced linearly from 2.5 to 0.

#### 4.1.4 The exploration and the exploitation phases

**Fig 2:**
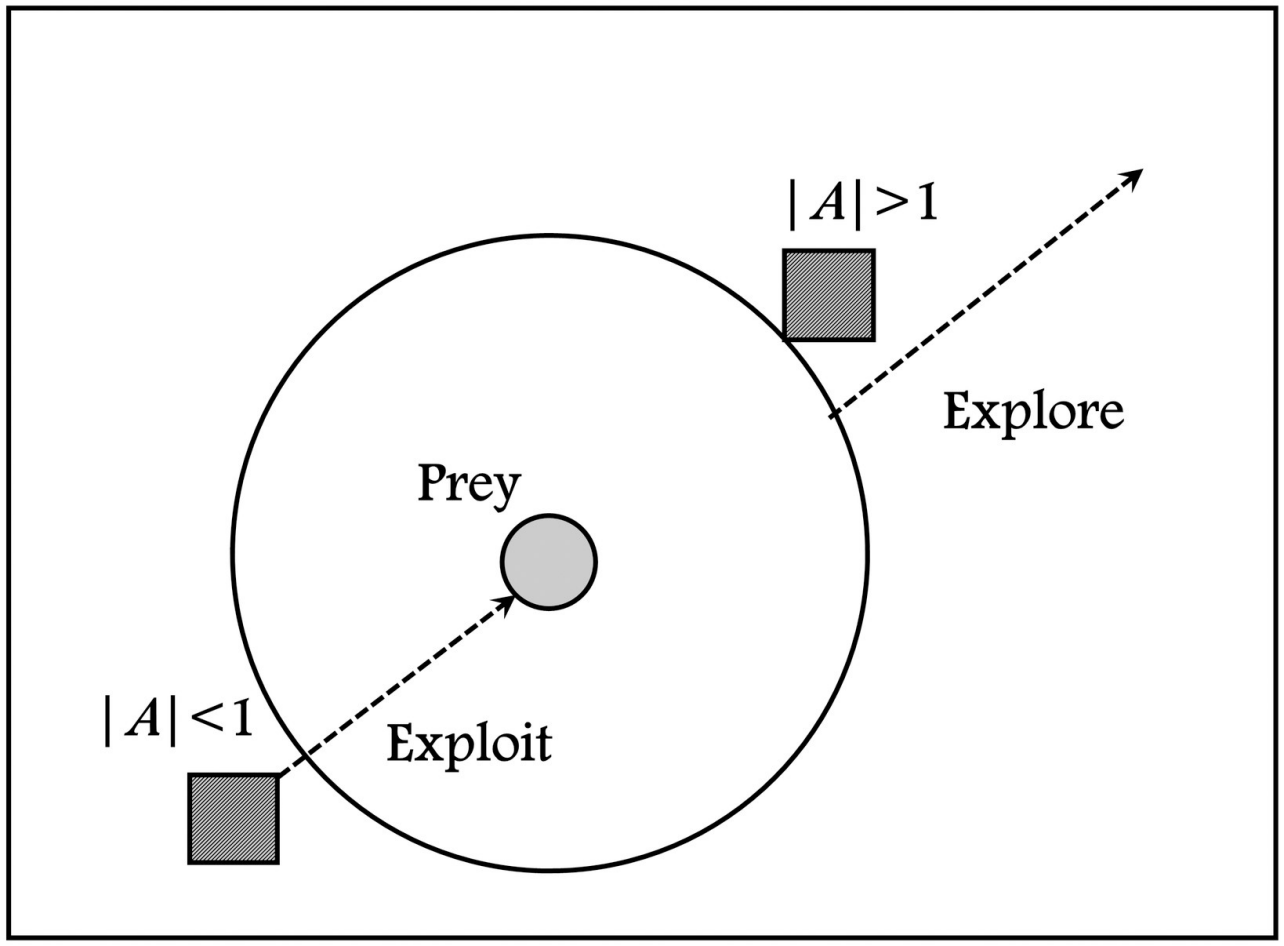
The effect of the vector *A* on the exploration and the exploitation phases.

The exploration phase is handled by the driver, chaser, and barrier solutions, while the exploitation phase is handled by the attacker solution. According to Eq 12. the vector $\vec{A}$ has a value in the range of [−1, 1], and the ChOA algorithm is compelled to move from the exploration to the exploitation phases based on this value. The impact of the vector $\vec{A}$ on the exploration and exploitation phases is depicted in Fig 2.

$$\begin{array}{c} Phase=\{\begin{array}{cc} \text{Exploration} & if\;\;|\vec{A}|>1\\ \text{Exploitation} & if\;\;|\vec{A}|<1 \end{array} \end{array}$$ (12)

#### 4.1.5 The solution updating process

The individuals in the population update their position based on the values of the four leaders’ individuals (attacker, driver, chaser, and barriers). This process can be formulated as follows.

$$\begin{array}{c} {\vec{X}}^{\left(t+1\right)}=\frac{\vec{X_{1}}+\vec{X_{2}}+\vec{X_{3}}+\vec{X_{4}}}{4} \end{array}$$ (13)

**Fig 3:**
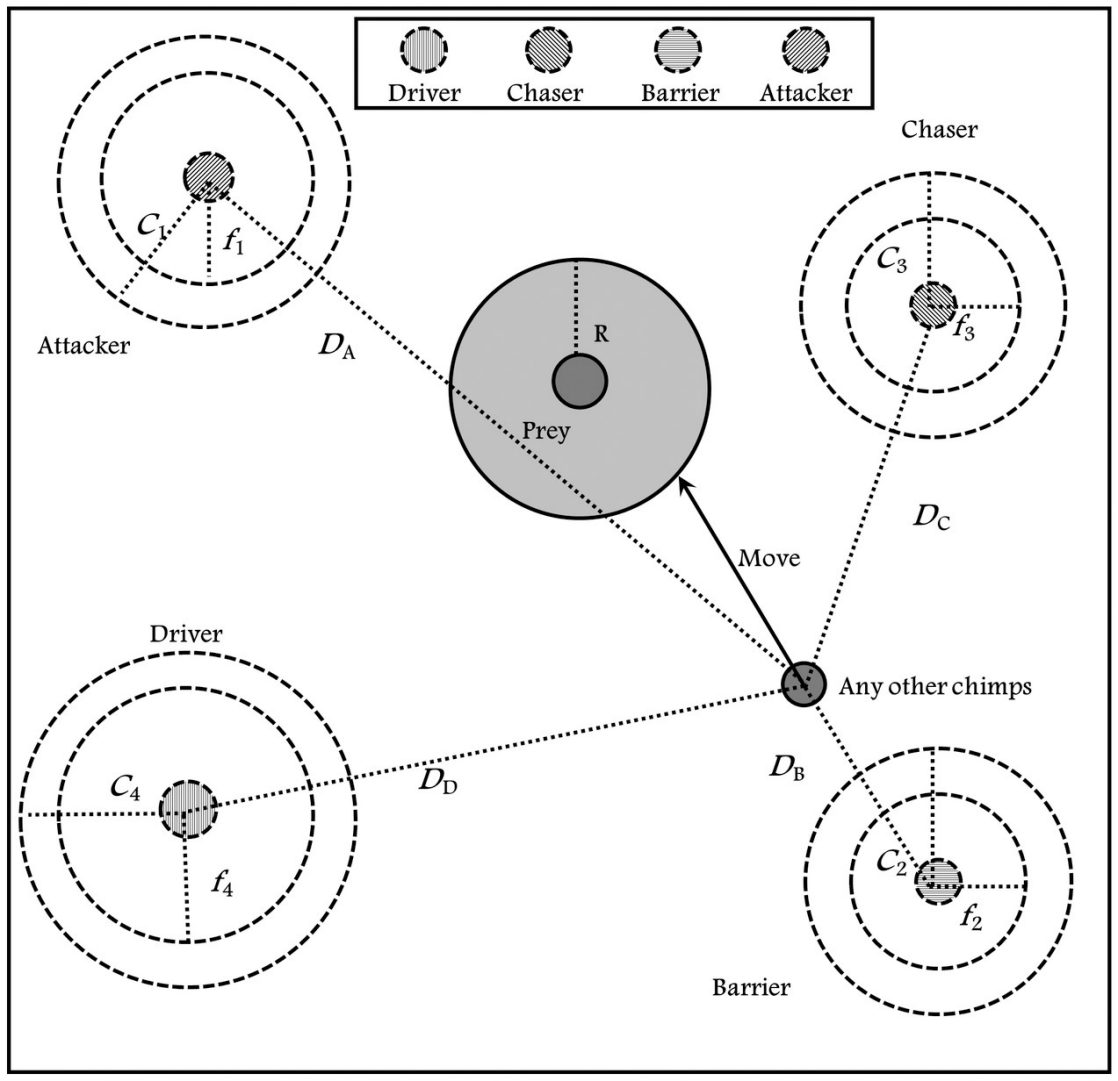
Individuals updating.

Where *t* is the current iteration, the vectors $\vec{X_{1}}$, $\vec{X_{2}}$, $\vec{X_{3}}$, and $\vec{X_{4}}$ are calculated in Eqs 5, 6, 7 and 8. Fig 3 shows the individuals updating process in the ChOA.

#### 4.1.6 The sexual motivation process

In the final stage of the hunting process, some chimps release their duties and they try to get the food randomly. This situation can be simulated in the ChOA to accelerate the convergence and avoid trapping in local optima. In the ChOA, the value of the parameter *μ* is responsible for switching between the normal updating position and the chaotic updating positions for all individuals in the population. This process can be formulated as follows.

$$\begin{array}{c} {\vec{X}}^{\left(t+1\right)}=\{\begin{array}{cc} \text{Equation}\;13 & if\;\;\mu <0.5\\ \text{Chaotic}\;\text{value} & if\;\;\mu \geq 0.5 \end{array} \end{array}$$ (14)

#### 4.1.7 The structure of the ChOA

The overall processes of the ChOA are presented in Algorithm 1 as follows.

**Algorithm 1**: The pseudocode ChOA algorithm

1: Set the initial values for the vectors $\vec{A}$, $\vec{M}$, $\vec{C}$, the coefficient *f*.

2: Set the initial value of the iteration numbers *t* ≔ 0.

3: Initialize the population *X* randomly, where $\vec{X_{i}^{\left(t\right)}}$, *i* = 1, …, *N*.

4: Calculate the fitness function of each individual $\vec{X_{i}^{\left(t\right)}}$.

5: Assign the attacker *X*_*A*_ as shown in Eq 5.

6: Assign the barrier *X*_*B*_ as shown in Eq 6.

7: Assign the chaser *X*_*C*_ as shown in Eq 7.

8: Assign the driver *X*_*D*_ as shown in Eq 8.

9: **repeat**

10:  **for** i = 1 to N **do**

11:   Generate a random number *r*_1_, and *r*_2_ ∈ [0, 1].

12:   Update the values of the vectors $\vec{M}$ as shown in Eq 11.

13:   Update the values of the vectors $\vec{C}$ as shown in Eq 10.

14:   Update the values of the coefficient *f*.

15:   Update the value of the vector $\vec{A}$ as shown in Eq 9.

16:   **if** (*μ* < 0.5) **then**

17:    **if** ($\vec{A}<1$) **then**

18:     Update the position of the current individual as shown in Eq 13. {**Exploitation phase**}

19:    **else**

20:     Select the individual randomly. {**Exploration phase**}

21:    **end if**

22:   **else**

23:    Update the individual based on the chaotic value.

24:   **end if**

25:  **end for**

26:  Update the values of the vectors $\vec{M}$, $\vec{A}$, $\vec{C}$ and the coefficient *f*.

27:  Update attacker *X*_*A*_ as shown in Eq 5.

28:  Update the barrier *X*_*B*_ as shown in Eq 6.

29:  Update the chaser *X*_*C*_ as shown in Eq 7.

30:  Update the driver *X*_*D*_ as shown in Eq 8.

31:  Set *t* = *t* + 1.

32: **until** Termination criteria satisfied.

33: Produce the overall individual.

### 4.2 The structure of the proposed MWChOA algorithm

In the standard ChOA, the positions of the other chimpanzees are only updated using the first four ChOA solutions: driver, chaser, attacker, and barrier. Instead, the four best selections attract the other chimps’ attention (driver, chaser, barrier, and attacker). Despite the fact that attackers can naturally predict the course of their prey’s evolution, there is no assurance that their strategy will always be the best because chimpanzees occasionally abandon their duties while hunting or continue to do so throughout the process. If the position of other chimps is updated based on the attackers, they may become stuck in the local optima and be unable to explore new parts of the search space since their solution space is extremely concentrated around the attacker’s solutions. The greatest alternatives have similar justifications as well (driver, chaser, barrier). To increase the algorithm’s convergence speed, we used three first three leader solutions (chasers, an attacker, and a barrier). Eqs 15, 16 and 17 are used instead of 5, 6, 7 and 8.

$$\begin{array}{c} \vec{D_{A}}=\left|\vec{C_{1}}\vec{X_{A}}-\vec{M_{1}}\vec{X}\right|,\\ \vec{X_{1}}=\left|\vec{X_{A}}-\vec{A_{1}}\vec{D_{A}}\right| \end{array}$$ (15)

$$\begin{array}{c} \vec{D_{B}}=\left|\vec{C_{2}}\vec{X_{B}}-\vec{M_{2}}\vec{X}\right|,\\ \vec{X_{2}}=\left|\vec{X_{B}}-\vec{A_{2}}\vec{D_{B}}\right| \end{array}$$ (16)

$$\begin{array}{c} \vec{D_{C}}=\left|\vec{C_{3}}\vec{X_{C}}-\vec{M_{3}}\vec{X}\right|,\\ \vec{X_{3}}=\left|\vec{X_{C}}-\vec{A_{3}}\vec{D_{C}}\right| \end{array}$$ (17)

Then, in order to hasten convergence and enhance exploration and exploitation, a position-weighted equation based on weights is created [88]. Use equations (1) through (3) to update additional chimpanzee locations (3). Other chimpanzees are essentially compelled to alter their positions in accordance with those of the pursuer, attacker, and barrier. In light of the preceding justifications, it becomes possible to come up with fresh approaches to changing other chimpanzees’ perspectives. The weighting strategy proposed below is based on the step size’s Euclidean distance.

$$\begin{array}{c} W_{1}=\frac{|\vec{X_{1}}|}{|\vec{X_{1}}\left|+\right|\vec{X_{2}}\left|+\right|\vec{X_{3}}|} \end{array}$$ (18)

$$\begin{array}{c} W_{2}=\frac{|\vec{X_{2}}|}{|\vec{X_{1}}\left|+\right|\vec{X_{2}}\left|+\right|\vec{X_{3}}|} \end{array}$$ (19)

$$\begin{array}{c} W_{3}=\frac{|\vec{X_{3}}|}{|\vec{X_{1}}\left|+\right|\vec{X_{2}}\left|+\right|\vec{X_{3}}|} \end{array}$$ (20)

where the learning rates from the attacker, barrier, and chaser are denoted by *W*1, *W*2 and *W*3 respectively. Additionally,|.| displays the Euclidean distance. But the position-weighted connection looks like this:

$$\begin{array}{c} {\vec{X}}^{\left(t+1\right)}=\frac{1}{W_{1}+W_{2}+W_{3}}\times \frac{W_{1}\vec{X_{1}}+W_{2}\vec{X_{2}}+W_{3}\vec{X_{3}}}{3} \end{array}$$ (21)

Instead of using Eq 13 in the traditional ChOA, the position-weighted relationship Eq 21 can be used in MWChOA. It should be clear that applying the appropriate learning rate is the primary distinction between Eq 21 and the conventional position-weighted relationship, Eq 13. Consequently, the relationship shown below is used.

$$\begin{array}{c} {\vec{X}}^{\left(t+1\right)}=\{\begin{array}{cc} \text{Equation}\;21 & if\;\;\mu <0.5\\ \text{Chaotic}\;\text{value} & if\;\;\mu \geq 0.5 \end{array} \end{array}$$ (22)

In other words, the attacker, barrier, and chaser define where the other chimpanzees will eventually be located at random: in a circle around the victim.

#### 4.2.1 The structure of the MWChOA

**Fig 4:**
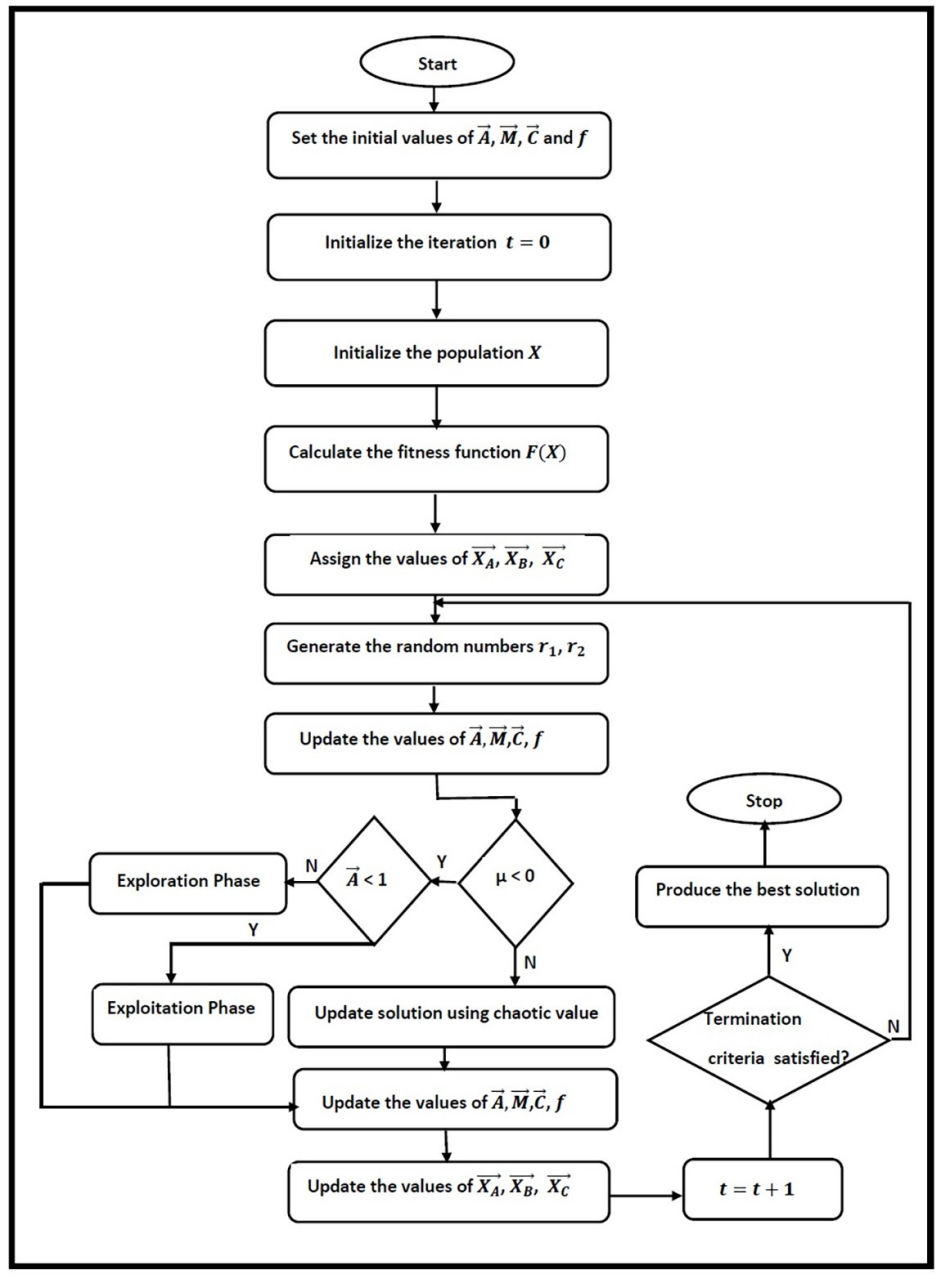
The main steps of the proposed MWChOA.

The overall processes of the MWChOA are presented in Algorithm 2 as follows. In the proposed algorithm, we reduce the number of the leader solutions from Four to Three to avoid trapping in local optima and balance between the exploration and exploitation during the search. The main steps of the proposed algorithm are shown in Fig 4.

**Algorithm 2**: The pseudocode MWChOA algorithm

1: Set the initial values for the vectors $\vec{A}$, $\vec{M}$, $\vec{C}$, the coefficient *f*.

2: Set the initial value of the iteration numbers *t* ≔ 0.

3: Initialize the population *X* randomly, where $\vec{X_{i}^{\left(t\right)}}$, *i* = 1, …, *N*.

4: Calculate the fitness function of each individual $\vec{X_{i}^{\left(t\right)}}$.

5: Assign the attacker *X*_*A*_ as shown in Eq 15.

6: Assign the barrier *X*_*B*_ as shown in Eq 16.

7: Assign the chaser *X*_*C*_ as shown in Eq 17.

8: **repeat**

9:  **for** i = 1 to N **do**

10:   Generate a random number *r*_1_, and *r*_2_ ∈ [0, 1].

11:   Update the values of the vectors $\vec{M}$ as shown in Eq 11.

12:   Update the values of the vectors $\vec{C}$ as shown in Eq 10.

13:   Update the values of the coefficient *f*.

14:   Update the value of the vector $\vec{A}$ as shown in Eq 9.

15:   **if** (*μ* < 0.5) **then**

16:    **if** ($\vec{A}<1$) **then**

17:     Update the position of the current individual as shown in Eq 21. {**Exploitation phase**}

18:    **else**

19:     Select the individual randomly. {**Exploration phase**}

20:    **end if**

21:   **else**

22:    Update the individual based on the chaotic value.

23:   **end if**

24:  **end for**

25:  Update the values of the vectors $\vec{M}$, $\vec{A}$, $\vec{C}$ and the coefficient *f*.

26:  Update attacker *X*_*A*_ as shown in Eq 15.

27:  Update the barrier *X*_*B*_ as shown in Eq 16.

28:  Update the chaser *X*_*C*_ as shown in Eq 17.

29:  Set *t* = *t* + 1.

30: **until** Termination criteria satisfied.

31: Produce the overall individual.

## 5 Experimental results

### 5.1 Simulation platform

All algorithms are tested in Matlab R2018a and run on a PC with an Intel(R) Core(TM) i7–9700 processor 3.00 GHz, 8 GB of RAM, and Windows 10.

### 5.2 Datasets

The proposed MWChOA is tested on Eleven benchmark datasets from the UCI repository [89]. Table 1 displays the chosen datasets and their attributes and classes.

**Table 1:** Datasets.

Classification datasets	Number of attributes	Number of classes
3-bits XOR	3	2
Balloon	4	2
Iris	4	3
Breast cancer	9	2
Heart	22	2
Hepatitis	19	2
Haberman	3	2
Liver	6	2
Ionosphere	34	2
Lung Cancer	56	2
pima	8	2

#### 5.2.1 XOR dataset

A well-known nonlinear benchmark problem is the N-bit XOR problem. The goal is to figure out how many “1”s there are in the input vector. The input vector’s XOR result should be returned; if the input vector has an odd number of “1 s,” the output should be “1.” The output is “0” if the input vector contains an even number of “1 s” [90]. There are three inputs, eight training test samples, and one output in the XOR dataset. To tackle this problem, we employ the 3–7-1 FNN structure

#### 5.2.2 Balloon dataset

This set of data is based on balloon inflation experiments conducted under a variety of situations. This data set has 16 instances and 16 test sets, each with four attributes: colour, size, action, and age, as well as four inputs and one output that shows whether the balloon is inflated or not [91]. To categorise this dataset, we utilise a neural network organised 4–9-1.

#### 5.2.3 Breast cancer dataset

William H. Wolbergby of the University of Wisconsin-Madison Hospital developed this dataset. The goal of this collection is to use photographs to determine whether a patient has cancer. This dataset’s classification and recognition are really important. The data collection contains 699 occurrences and 9 attributes, including clump thickness, cell size uniformity, cell shape uniformity, and edge adhesion. The output is 2 if the classification recognition result is a benign tumour, and 4 if the classification recognition result is a malignant tumour [92]. As a result, the 9–19-1 FNNs architecture is used to classify this dataset.

#### 5.2.4 Iris dataset

The iris data set is used to categorise various iris types, which are split into three categories: Setosa, Versicolor, and Virginica. There are three outputs, in other words. There are 150 examples with four different characteristics: sepal length, sepal width, petal length, and petal width [93], totaling four inputs. As a result, for training and solving, this data set employs FNNs with a structure of 4–9-3.

#### 5.2.5 Heart dataset

This data collection is utilised for cardiac single proton computed tomography (SPECT) image diagnosis recognition and classification. The classification results indicate if the output patient is normal or abnormal [94]. All features are in binary format. This data set is really complex. It has 22 features and 22 inputs, including 80 data examples and 187 test cases. For training, we create a neural network with a structure of 22–45–1. In Table 2, the experimental findings are displayed. The classification set’s hardest training dataset is the heart.

**Table 2:** The balloon dataset’s statistical results.

Algorithm	AVE	STD	CR (%)
MWChOA	8.10*e*^−17^	2.33*e*^−16^	**100.00**
WChOA	4.76*e*^−04^	0.001008	**100.00**
ChOA	8.52*e*^−04^	0.001402	75
HHO	9.82*e*^−07^	3.09*e*^−06^	**100.00**
GWO	9.38*e*^−15^	2.81*e*^−14^	**100.00**
ABC	2.08*e*^−16^	2.17*e*^−16^	**100.00**
HS	0.004628	0.006588	100.00
GGSA	0.006771	0.006925	100.00
DE	2.67*e*^−07^	7.34*e*^−07^	**100.00**
PSO	0.000585	0.000749	100.00
GA	**5.08*e*^−24^**	**1.06*e*^−23^**	**100.00**
ACO	0.004854	0.007760	**100.00**
ES	0.019055	0.170260r	**100.00**
PBIL	2.49*e*^−05^	5.27*e*^−05^	**100.00**
SSA	6.394*e*^−05^	9.958^−05^	**100.00**
SSO	5.2874*e*^−13^	1.2745*e*^−12^	**100.00**

#### 5.2.6 Hepatitis dataset

the hepatitis database categorises whether the output patient is alive or dead [95]. 19 characteristics and 19 inputs are present. We build a neural network with the structure 19–39-1 for training FNN.

#### 5.2.7 Haberman dataset

The dataset includes data from a research on the prognosis of breast cancer patients who underwent surgery that was carried out at the University of Chicago’s Billings Hospital between 1958 and 1970 [96]. The classification results show whether the patient died within 5 years or survived for 5 years or more. this data set employs FNNs with a structure of 3–7-1.

#### 5.2.8 Liver dataset

All of the blood tests that make up the attributes of this dataset are thought to be sensitive to liver diseases that may be caused by drinking too much alcohol [97]. One single male person’s record is contained in each line of the dataset. We create a neural network with a structure of 6–13–1.

#### 5.2.9 Ionosphere dataset

This dataset consists of radar data that was gathered by a device in Goose Bay, Labrador [98]. With a total transmission power of around 6.4 kilowatts, this system comprises of a phased array of 16 high-frequency antennas. In the ionosphere, free electrons were the intended targets (good or bad). A structure of some kind in the ionosphere can be seen in “good” radar signals. The ones that don’t have their transmissions cut through the ionosphere are considered “bad” returns. The structure of FNN is 34–69-1.

#### 5.2.10 Lung cance dataset

The Lung dataset is a large dataset that includes all of the study data accessible for analysis on lung cancer screening, incidence, and death [99]. We build a neural network with the structure 56–113-1 for training FNN.

#### 5.2.11 Pima dataset

The National Institute of Diabetes and Digestive and Kidney Diseases is the original source of this dataset [100]. The dataset’s goal is to diagnostically classify whether or not a patient has diabetes. The 9–19-1 FNNs structure used to classify this dataset.

### 5.3 Parameter setting

The parameters setting of the proposed algorithm and other algorithms are shown in Tables 3 and 4.

**Table 3:** The initial parameters of algorithms.

Algorithm	Parameter	Value
MWCHOA	*m*	Gauss/mouse map
	*r*_1_, *r*_2_	random number
	Population size	XOR and Balloon get 50, while the rest get 200
	The maximum number of iterations	250
CHOA	*m*	chaotic maps
	*r*_1_, *r*_2_	random number
	Population size	XOR and Balloon get 50, while the rest get 200
	The maximum number of iterations	250
HHO	*β*	1.5
	Population size	XOR and Balloon get 50, while the rest get 200
	The maximum number of iterations	250
GWO	*ρ*	decreased linearly from 2 to 0
	*α*	
	Population size	XOR and Balloon get 50, while the rest get 200
	The maximum number of iterations	250
ABC	Limit	*D*
	Population size	XOR and Balloon get 50, while the rest get 200
	The maximum number of iterations	250
HS	Harmony memory accepting rate	0.75
	Pitch adjusting rate	0.7
	Population size	XOR and Balloon get 50, while the rest get 200
	The maximum number of iterations	250
GGSA	Accelerating coefficient $\left(c_{1}^{\prime }\right)$	(−2*t*^3^/*T*^3^) + 2
	Accelerating coefficient $\left(c_{2}^{\prime }\right)$	(2*t*^3^/*T*^3^)
	Gravitational constant(*G*_0_)	1
	Coefficient of decrease constant (*α*)	20
	Population size	XOR and Balloon get 50, while the rest get 200
	The maximum number of iterations	250
DE	Scaling factor(*F*)	0.5
	Crossover Probability (*CR*)	0.5
	Population size	XOR and Balloon get 50, while the rest get 200
	The maximum number of iterations	250
PSO	Topology	Fully connected
	Cognitive constant (*c*_1_)	1
	Social constant(*c*_2_)	1
	Inertia constant *ω*	0.3
	Population size	XOR and Balloon get 50, while the rest get 200
	The maximum number of iterations	250
GA	Topology	Real coded
	Selection	Roulette wheel
	Crossover	Single point (probability = 1)
	Mutation	Uniform (probability = 0.01)
	Population size	XOR and Balloon get 50, while the rest get 200
	The maximum number of generations	250

**Table 4:** The initial parameters of algorithms.

Algorithm	Parameter	Value
ACO	Initial pheromone (*τ*)	1*e*^−06^
	Pheromone update constant (*Q*)	20
	Pheromone constant (*q*)	1
	Global pheromone decay rate (*P*_*g*_)	0.9
	Local pheromone decay rate (*P*_*t*_)	0.5
	Pheromone sensitivity (*α*)	1
	Visibility sensitivity (*β*)	5
	Population size	XOR and Balloon get 50, while the rest get 200
	The maximum number of iterations	250
ES	*λ*	10
	*σ*	1
	Population size	XOR and Balloon get 50, while the rest get 200
	The maximum number of iterations	250
PBIL	Learning rate	0.05
	Good population member	1
	Bad population member population member	0
	Elitism parameter	1
	Mutational probability	0.1
	Population size	XOR and Balloon get 50, while the rest get 200
	The maximum number of iterations	250
SSA	Population size	XOR and Balloon get 50, while the rest get 200
	The maximum number of generations	250
SSO	Population size	XOR and Balloon get 50, while the rest get 200
	The maximum number of iterations	250

### 5.4 The MWChOA for training FNN

- **The initial population**. The initial population contains weights and biases, which are generated randomly as shown in Eq 23.
  $$\begin{array}{c} \vec{V}=\left\{\vec{W},\vec{\theta }\right\}=\left\{W_{11},W22,\ldots ,W_{nn},\theta_{1},\theta_{2},\ldots ,\theta_{n}\right\} \end{array}$$ (23)
- **The fitness function**. The proposed algorithm uses the mean square error (MSE) to evaluate the obtained results by subtracting the desired results from the actual results as shown in Eq 24. The main goal is to minimize the MSE to obtain the best solution as shown in Eq 26.
  $$\begin{array}{c} MSE=\sum_{i=1}^{m}{\left(o_{i}^{k}-d_{i}^{k}\right)}^{2} \end{array}$$ (24)
  where $d_{i}^{k}$ is the desired output of the *i*th input unit when the *k*th training sample is utilised, and $o_{i}^{k}$ is the actual output of the *i*th input unit when the *k*th training sample occurs in the input, where *m* is the number of outputs. In datasets, there is always more than one training sample. As a result, all training samples should be checked for FNN. In these situations, the MSE average over all training samples is as follows:
  $$\begin{array}{c} \bar{MSE}=\sum_{k=1}^{s}\frac{\sum_{i=1}^{m}{\left(o_{i}^{k}-d_{i}^{k}\right)}^{2}}{S} \end{array}$$ (25)
  The number of training samples is *m*, and the number of outputs is *s* [59].
  $$\begin{array}{c} Minimize\;F\bar{\left\{V\right\}}=\bar{MSE} \end{array}$$ (26)
- **The hidden layer nodes *h***. The structure of FNNs is also important in the experimental setting, and we utilise the number of hidden layer neurons for datasets as follows:
  $$\begin{array}{c} h=2*n+1 \end{array}$$ (27)
  where *n* denotes the number of inputs and *h* denotes the number of hidden nodes.
- **The evaluated metrics**. Classification metrics assess the effectiveness of the proposed algorithm for training FNN and determine how accurate the classification is [101].
  **Accuracy** Shows how many cases are completely and correctly classified. It is derived by dividing the total number of predictions by the number of accurate predictions. It is calculated by Eq 28.
  $$\begin{array}{c} Accuracy=\frac{T_{p}+T_{N}}{T_{p}+T_{N}+F_{N}+F_{N}} \end{array}$$ (28)
  Where true positive *T*_*p*_ is a positive-class sample that has been correctly categorised. False positive *F*_*p*_ sample is one that should have been labelled as negative but was instead classified as positive. True Negative *T*_*N*_ refers to a correctly classified negative-class sample. false negative *F*_*N*_ sample is one that should have been labelled as positive but was incorrectly classified as negative.
  **Recall** The true positive rate (TPR), hit rate, or recall of a classifier represents the proportion of correctly identified positive samples to the total number of positive samples and is calculated using Eq 29.
  $$\begin{array}{c} Recall=\frac{T_{p}}{T_{p}+F_{N}} \end{array}$$ (29)
  **Precision** represents the proportion of accurately identified positive samples to the total number of positive expected samples as mentioned in Eq 30.
  $$\begin{array}{c} Precision=\frac{T_{P}}{F_{P}+T_{P}} \end{array}$$ (30)
  **The *F*_1_-score** is the harmonic mean of precision and recall. F-measure values range from zero to one, with higher values indicating better classification ability and is calculated using Eq 31.
  $$\begin{array}{c} F_{1}-score=\frac{2T_{p}}{2T_{p}+F_{p}+F_{N}} \end{array}$$ (31)

### 5.5 Time complexity of the proposed MWChOA

The time complexity of the proposed algorithm is calculated based on the population size *N*, the problem dimension *n* and the maximum number of iterations *t* as follows.

- **Initialize the parameters**. The time complexity for initializing the parameters such as $\vec{A}$, $\vec{M}$, $\vec{C}$, the coefficient *f* is constant *C*.
- **Initialize the population**. The population contains *N* solutions and *n* variables. The time complexity to generate the initial population is *O*(*N* × *n*).
- **Update the Solutions**. The time complexity to update the solutions in the population is *O*(*N* × *n*).
- **Evaluate the fitness function**. The time complexity to evaluate the initial population is *O*(*N* × *n*).

The overall time complexity is *O*(*C* × *t* + *N* × *n* × *t*), where *t* is the maximum iteration number.

### 5.6 Results and discussion

MWChOA is used to train FNN and the results are compared to 16 algorithms such as GWO [86], ABC [102], HS (Harmony Search) [103], GGSA (Gbest-guided Gravitational Search Algorithm) [104], DE (Differential Evolution) [105], PSO [86], GA [86], ACO [86], ES [86], PBIL [86], SSA [106], and SSO [107].

Table 5 shows the structure of the used FNN. In the following subsections, we reported the results of the proposed algorithm and the other algorithms.

**Table 5:** FNN structure for each dataset.

Classification datasets	Number of attributes	FNN structure
3-bits XOR	3	3–7–1
Balloon	4	4–9–1
Iris	4	4–9–3
Breast cancer	9	9–19–1
Heart	22	22–45–1
Hepatitis	19	19–39–1
Haberman	3	3–7–1
Liver	6	6–13–1
Ionosphere	34	34–69–1
Lung Cancer	56	56–113–1
pima	9	8–17–1

### 5.7 The performance of the proposed MWChOA algorithm

The proposed MWChOA algorithm is a modified version of the standard ChOA and the WChOA. In order to verify it efficiency, we compare it against these two algorithms by calcuating the four evaluation metrics (accuracy, precision, recall and F1-score). The results are reported in Tables 6 and 7, the overall best solution is reported in **bold text**.

**Table 6:** The accuracy and recall of the proposed MWChOA algorithm and the other algorithms.

	Accuracy			Recall		
Datasets	ChOA	WChOA	MWChOA	ChOA	WChOA	MWChOA
3-bits XOR	25.00	87.50	**100.00**	0.3333	0.7857	**1**
Balloon	75.00	100.00	**100.00**	0.7021	**1**	**1**
Iris	39.30	41.33	**92.90**	0.3803	0.6392	**0.9505**
Breast cancer	88.00	90.00	**99.00**	0.7931	0.9137	**1**
Heart	66.25	70.00	**88.75**	0.6044	0.7564	**0.9104**
Hepatitis	94.20	98.10	**99.40**	0.9526	**1**	**1**
Haberman	70.50	72.88	**74.20**	0.5760	0.6229	**0.6492**
Liver	98.00	99.00	**100.00**	1	**1**	**1**
Lonosphere	85.50	92.60	**99.40**	0.8069	0.9431	**1**
Lung Cancer	53.33	59.40	**81.30**	0.5458	0.6094	**0.8671**
Pima	71.00	74.60	**77.47**	0.6801	0.7203	**0.7547**

**Table 7:** The precision and F1-score of the proposed MWChOA algorithm and the other algorithms.

	Precision			F1-score		
Datasets	ChOA	WChOA	MWChOA	ChOA	WChOA	MWChOA
3-bits XOR	0.3750	0.6251	**1**	0.2857	0.7273	**1**
Balloon	0.7083	**1**	**1**	0.6970	**1**	**1**
Iris	0.4023	0.5237	**0.9018**	0.3961	0.6081	**0.9295**
Breast cancer	0.8714	0.9364	**1**	0.8732	0.9047	**1**
Heart	0.5753	0.7136	**0.9107**	0.5803	0.7208	**0.8753**
Hepatitis	0.9419	0.9806	**0.9935**	0.9701	0.9902	**0.9968**
Haberman	0.5472	**0.5746**	0.5637	0.5449	0.5777	**0.5991**
Liver	0.9839	0.9971	**1**	0.9927	0.9983	**1**
Lonosphere	0.8547	0.9259	**0.9943**	0.9217	0.9615	**0.9971**
Lung Cancer	0.5313	0.5938	**0.8125**	0.6939	0.7451	**0.8966**
Pima	0.6792	0.7098	**0.7227**	0.6777	**0.8335**	0.7327

**Fig 5:**
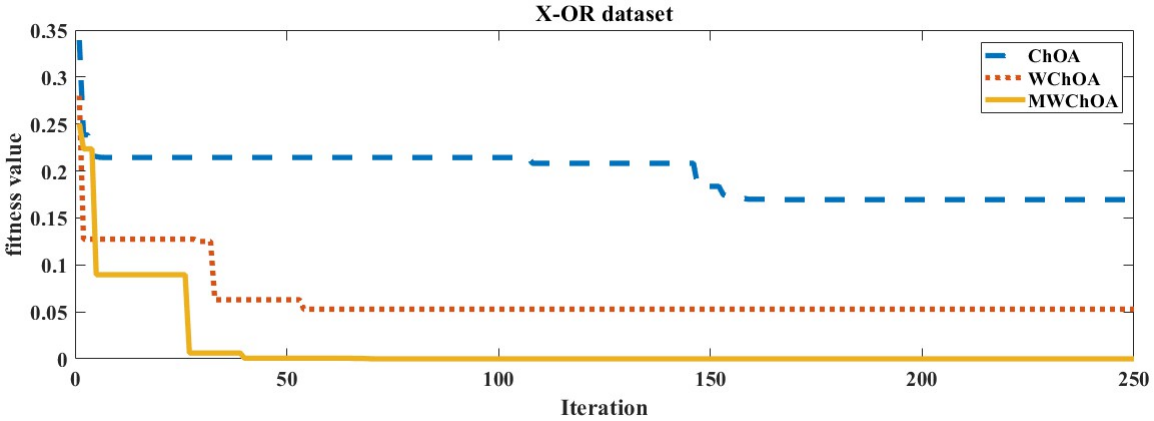
Convergence curves of algorithms for XOR.

**Fig 6:**
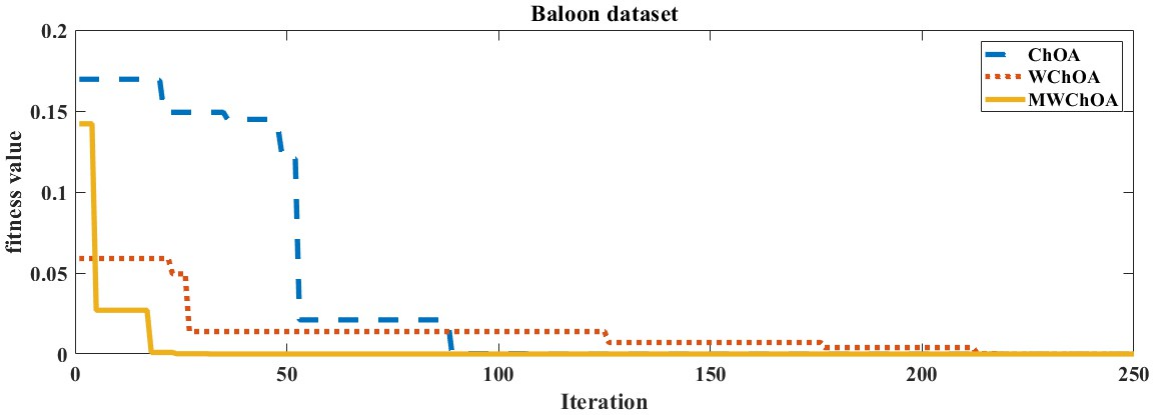
Convergence curves of algorithms for balloon.

**Fig 7:**
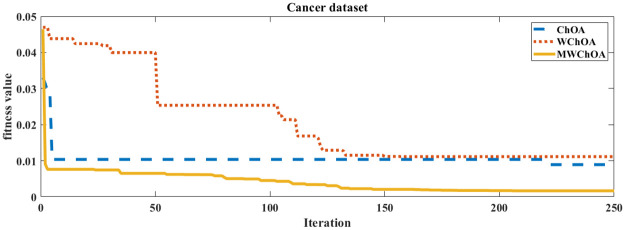
Convergence curves of algorithms for cancer.

**Fig 8:**
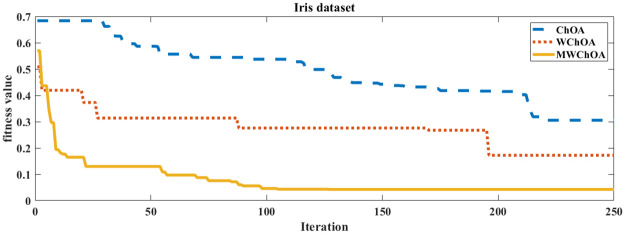
Convergence curves of algorithms for iris.

**Fig 9:**
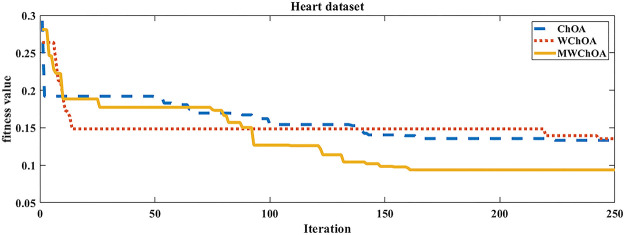
Convergence curves of algorithms for heart.

**Fig 10:**
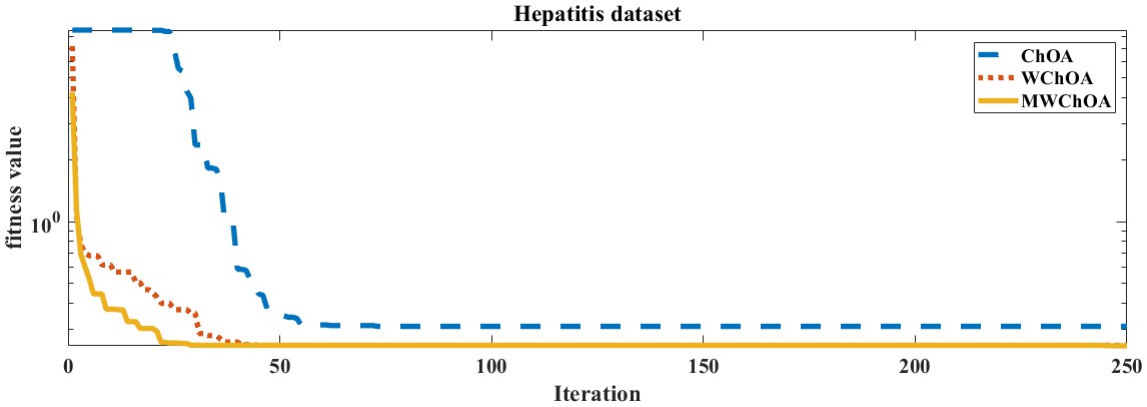
Convergence curves of algorithms for hepities.

**Fig 11:**
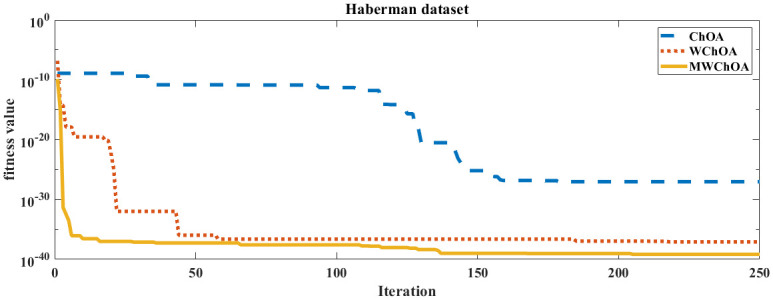
Convergence curves of algorithms for heberman.

**Fig 12:**
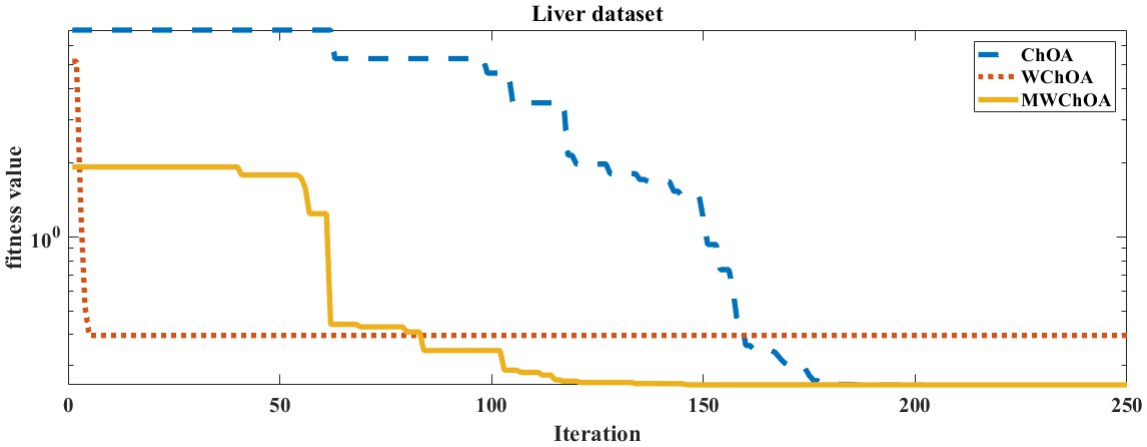
Convergence curves of algorithms for liver.

**Fig 13:**
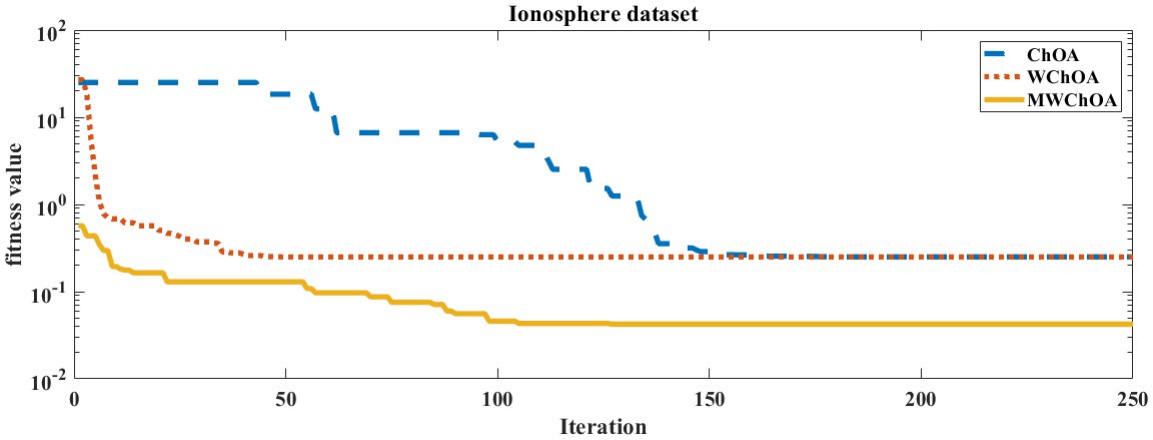
Convergence curves of algorithms for ionosphere.

**Fig 14:**
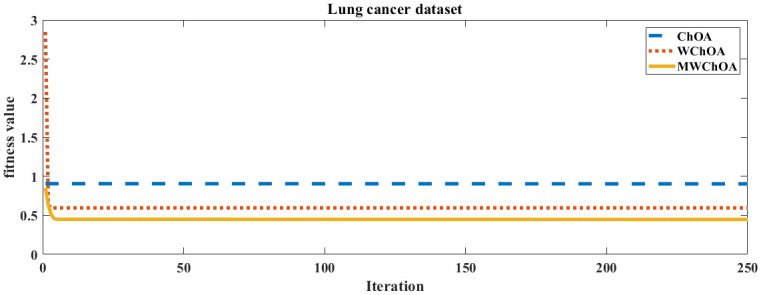
Convergence curves of algorithms for lung cancer.

**Fig 15:**
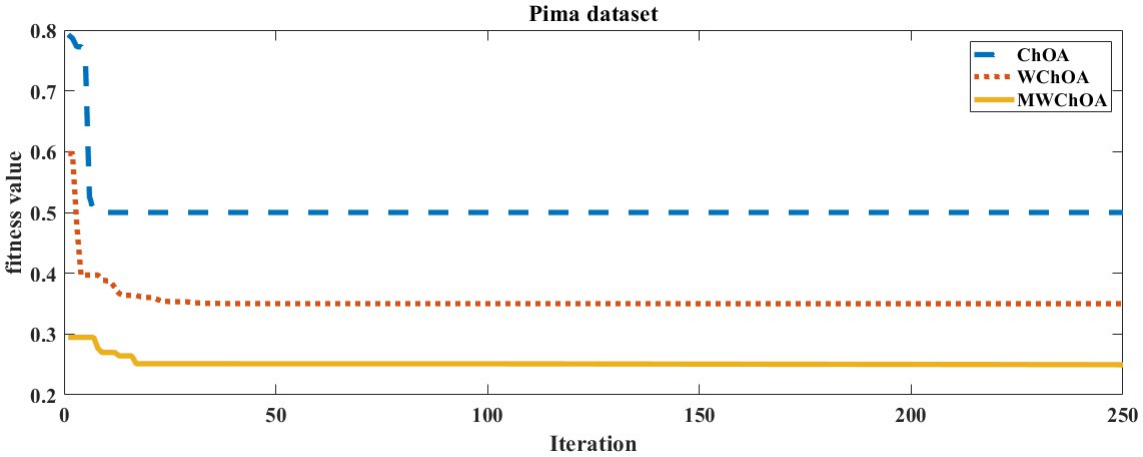
Convergence curves of algorithms for pima.

Also, we plot the convergence curve of the proposed algorithm and the ChOA and WChoA to ensure the efficiency of the proposed algorithm as shown in Figs 5–15. In these figures, we plot the relationship between the iterations and the fitness function. The solid line represents the results of the proposed MWChOA, the dotted line represents the WChOA algorithm and the dashed line represents the results of the standard ChOA algorithm. The results in all figures show that the proposed MWChOA is outperform the other algorithms and it converges faster than the other two algorithms.

### 5.8 The comparison between MWChOA and other algorithms

We applied another experiment to test the efficiency of the proposed MWChOA algorithm on the most used Five datasets such as XOR, balloon, breast cancer iris, and heart by comparing it against 16 SI algorithms such as GWO [86], ABC [102], HS (Harmony Search) [103], GGSA (Gbest-guided Gravitational Search Algorithm) [104], DE (Differential Evolution) [105], PSO [86], GA [86], ACO [86], ES [86], PBIL [86], SSA [106], and SSO [107].

The average (AVE), the standard deviations (STD), and the classification rate (CR%) are reported in Tables 8–11 for all algorithms after 10 runs. The overall results are reported in **bold text**. Also, the AVE, STD, and CR are plotted for all algorithms in Figs 16–30. The results in the tables and figures show that the proposed algorithm produces good results in most cases.

**Fig 16:**
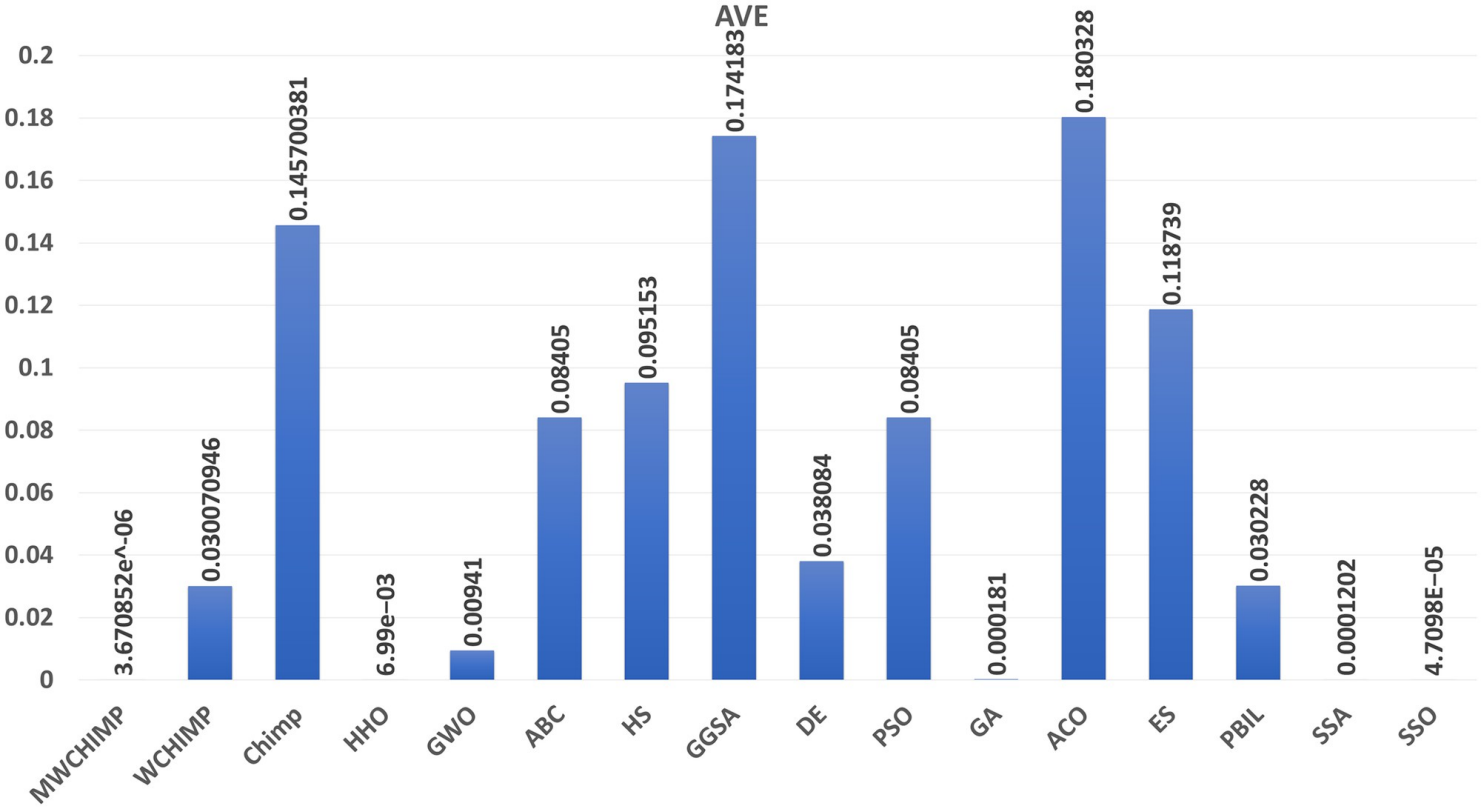
The AVE of algorithms for XOR.

**Fig 17:**
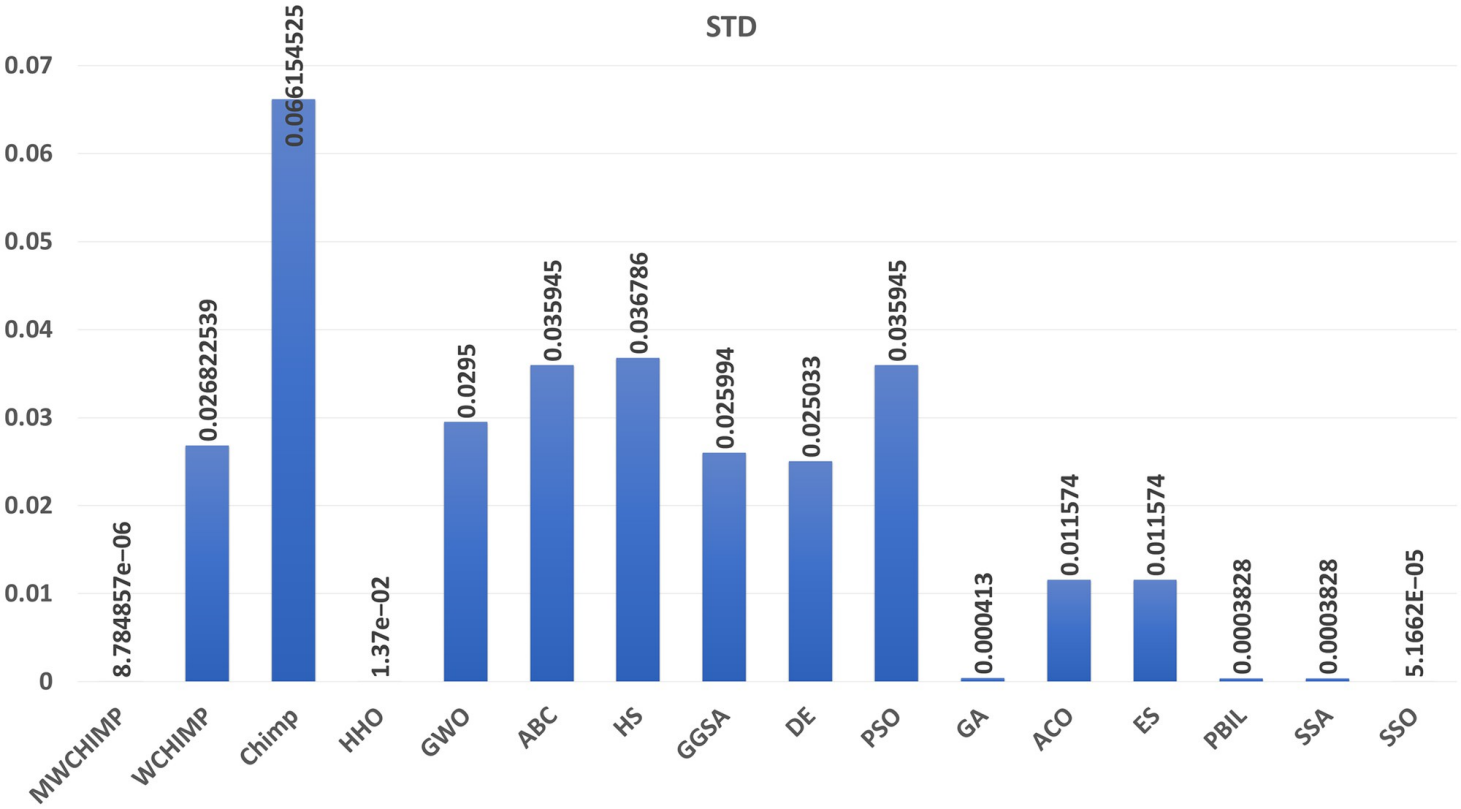
The STD of algorithms for XOR.

**Fig 18:**
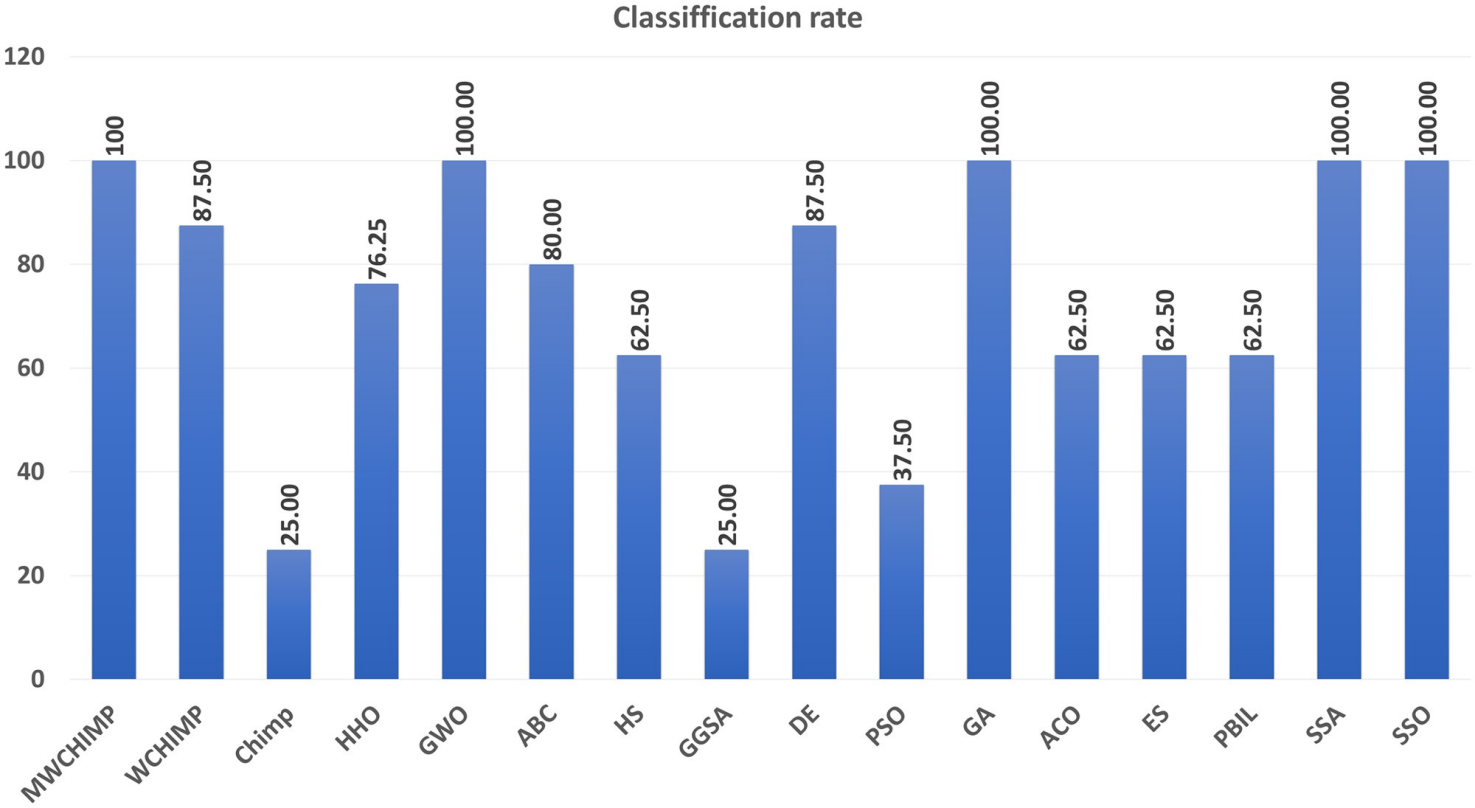
The classification rate of algorithms for XOR.

**Fig 19:**
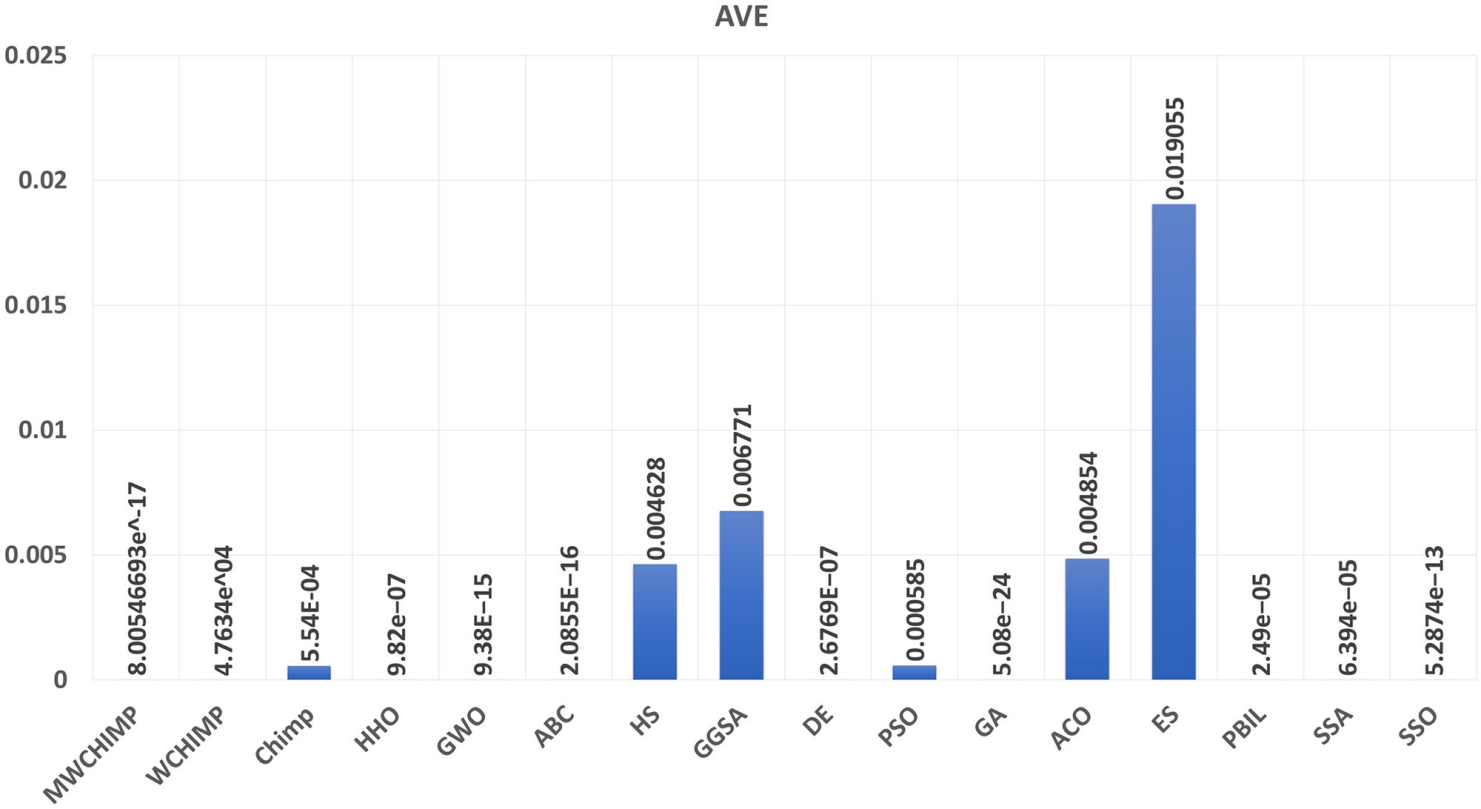
The AVE of algorithms for BALLOON.

**Fig 20:**
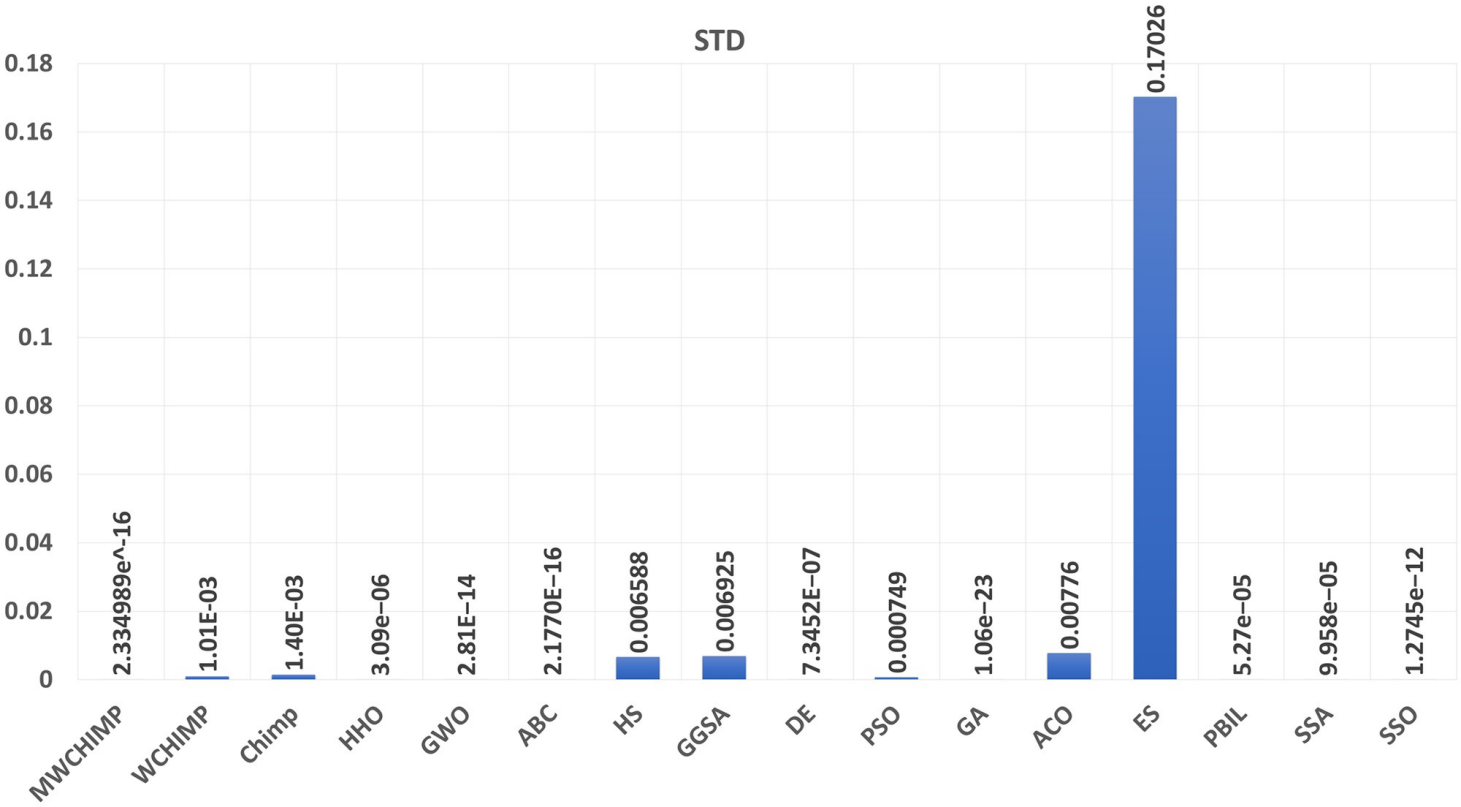
The STD of algorithms for BALLOON.

**Fig 21:**
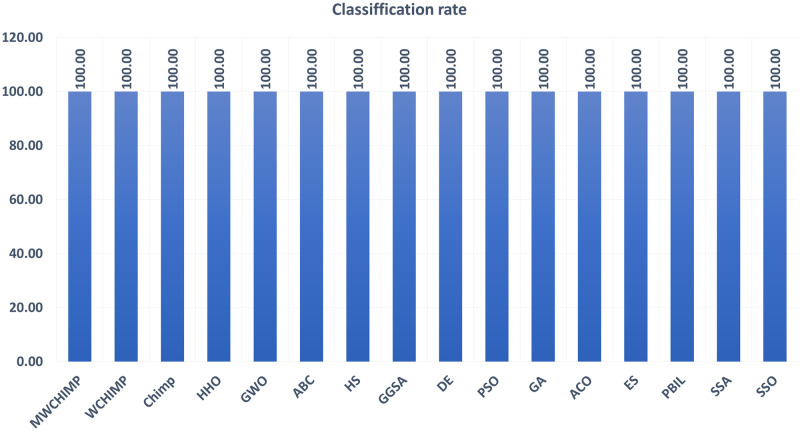
The classification rate of algorithms for BALLOON.

**Fig 22:**
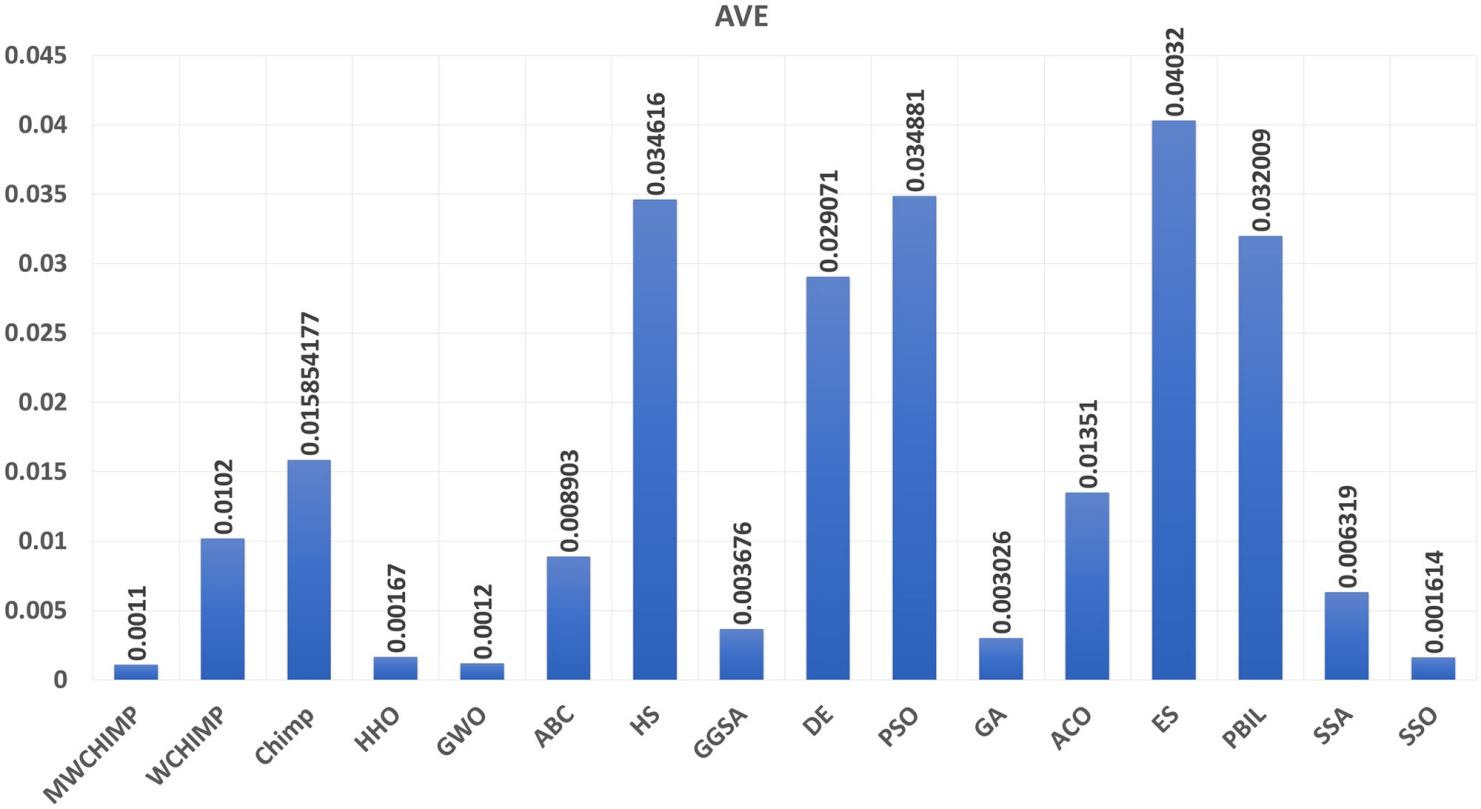
The AVE of algorithms for cancer.

**Fig 23:**
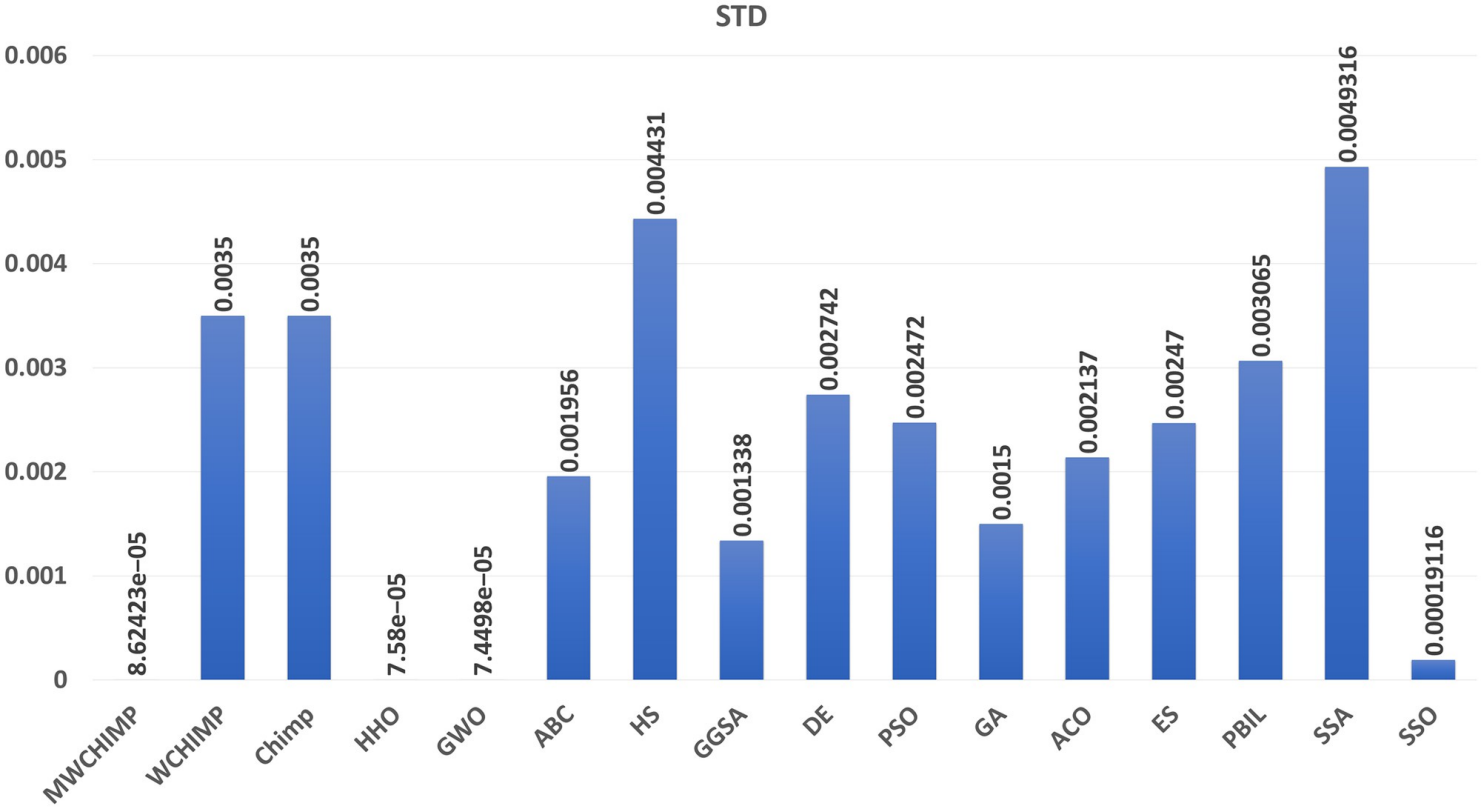
The STD of algorithms for cancer.

**Fig 24:**
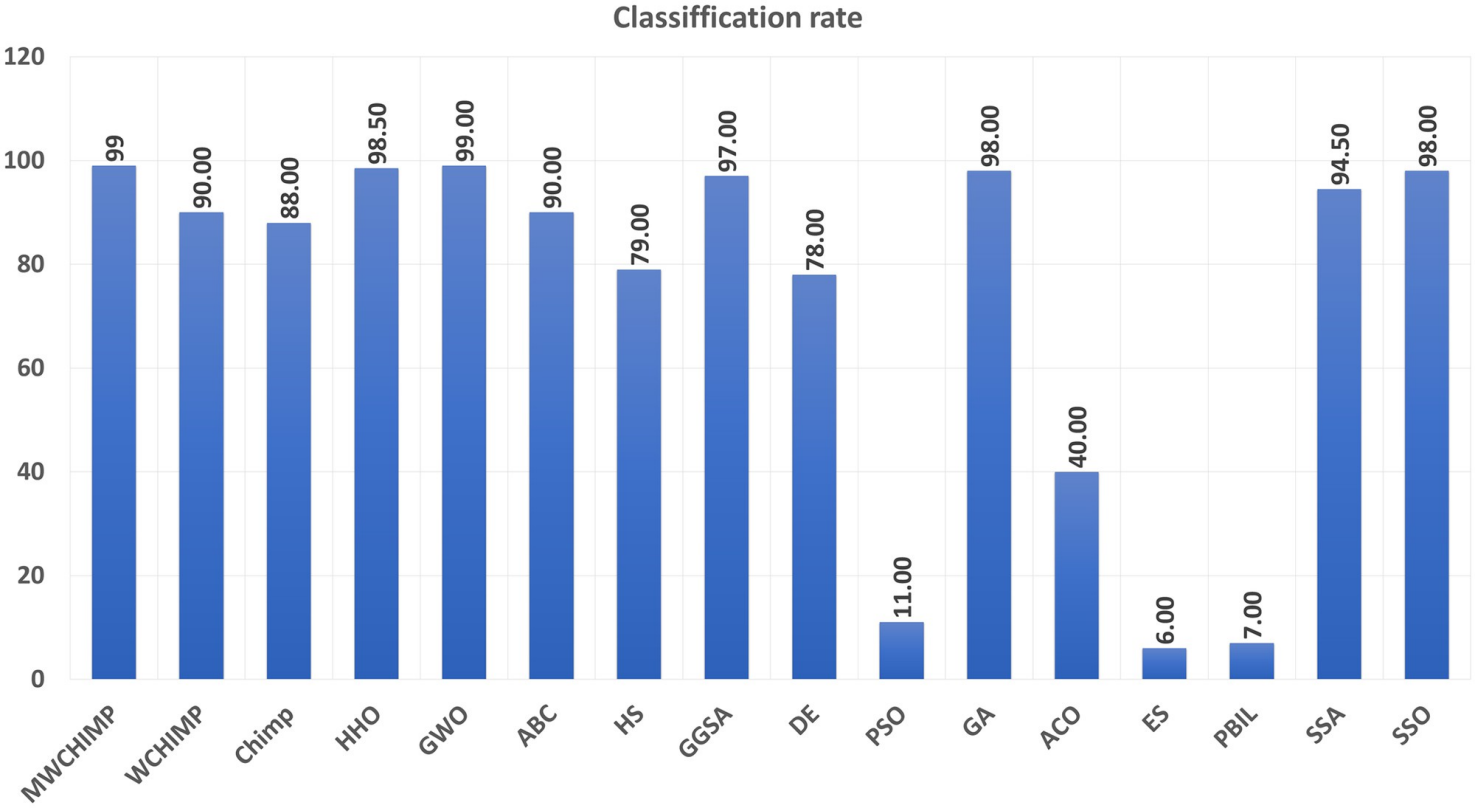
The classification rate of algorithms for cancer.

**Fig 25:**
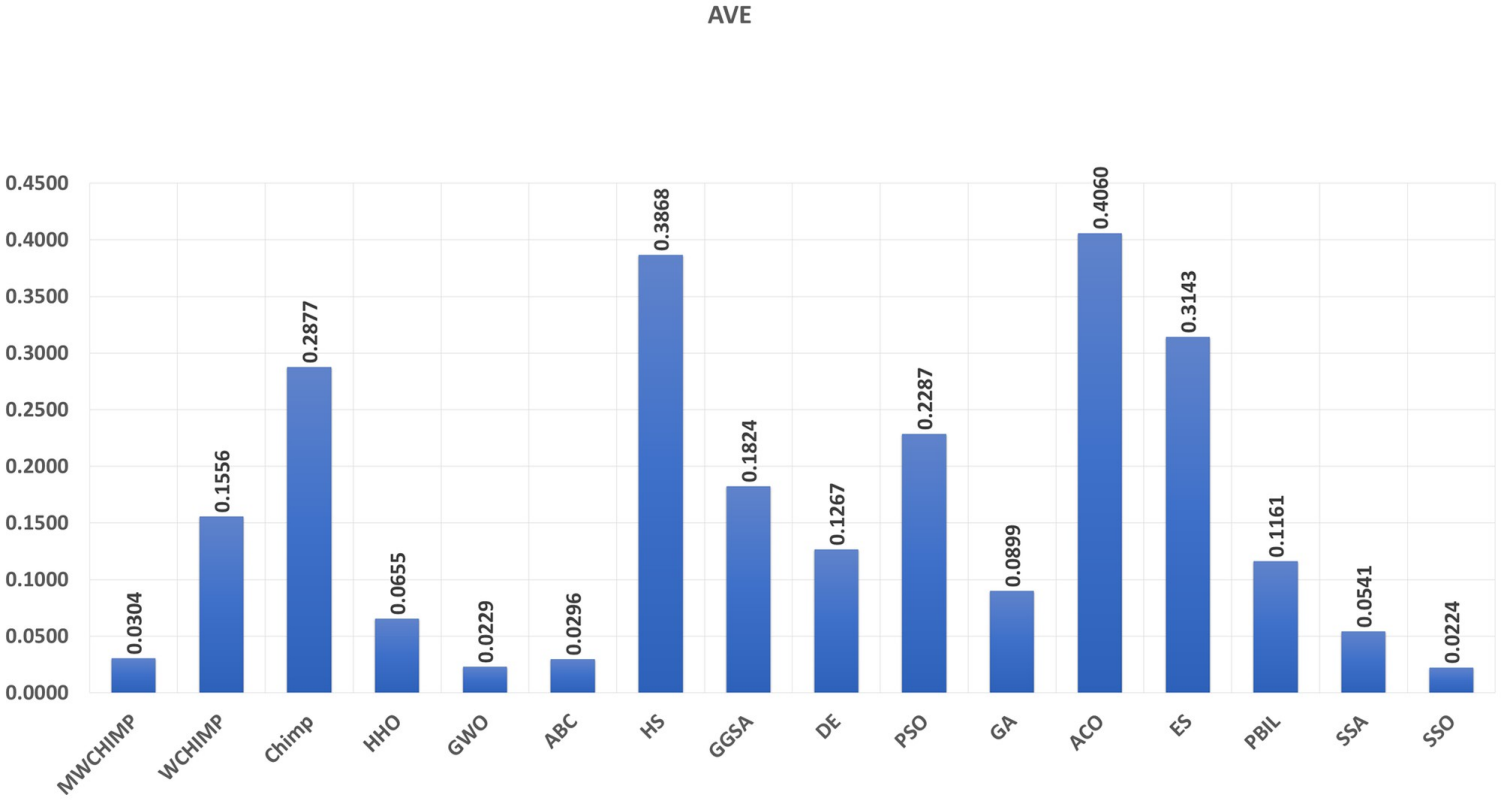
The AVE of algorithms for IRIS.

**Fig 26:**
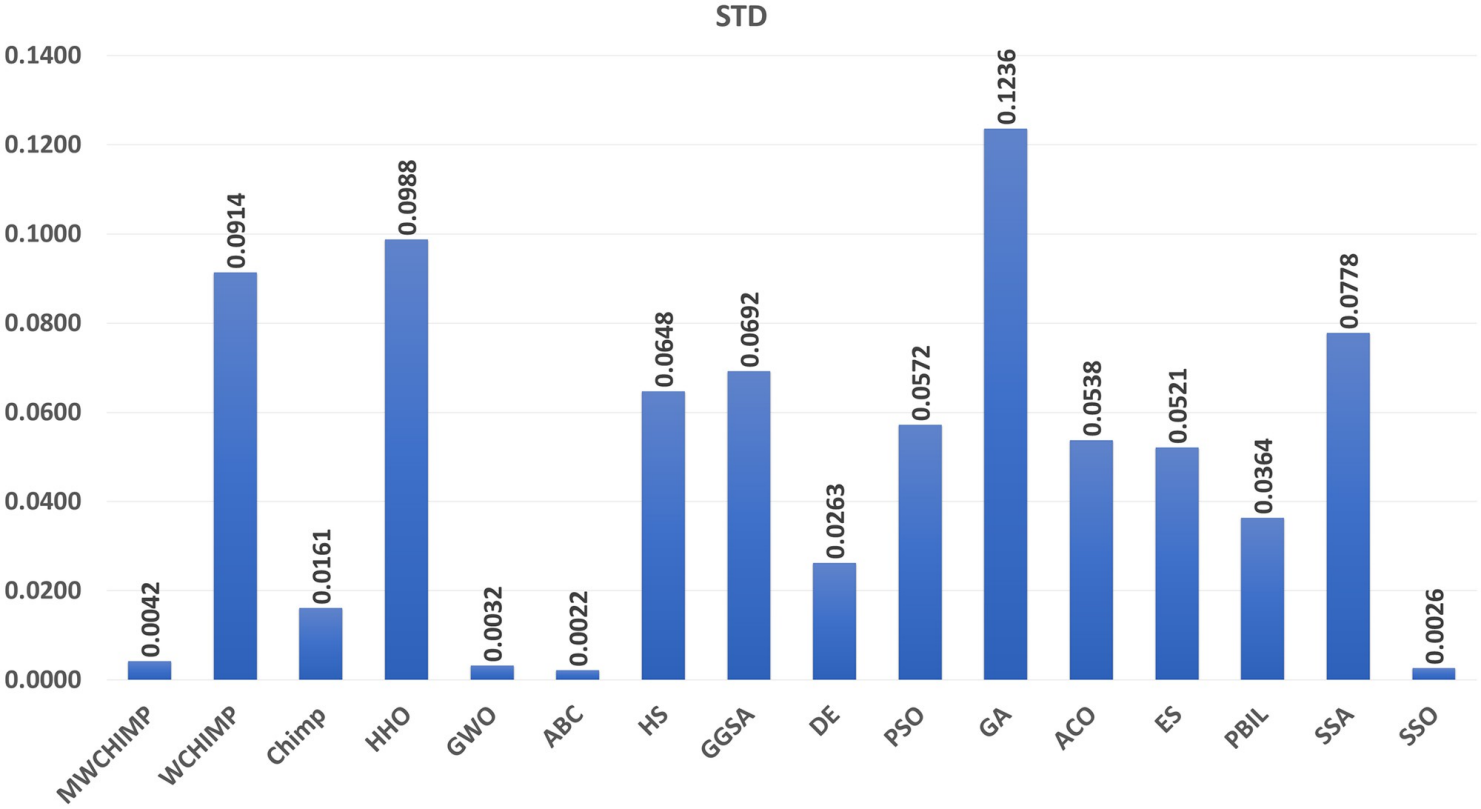
The STD of algorithms for IRIS.

**Fig 27:**
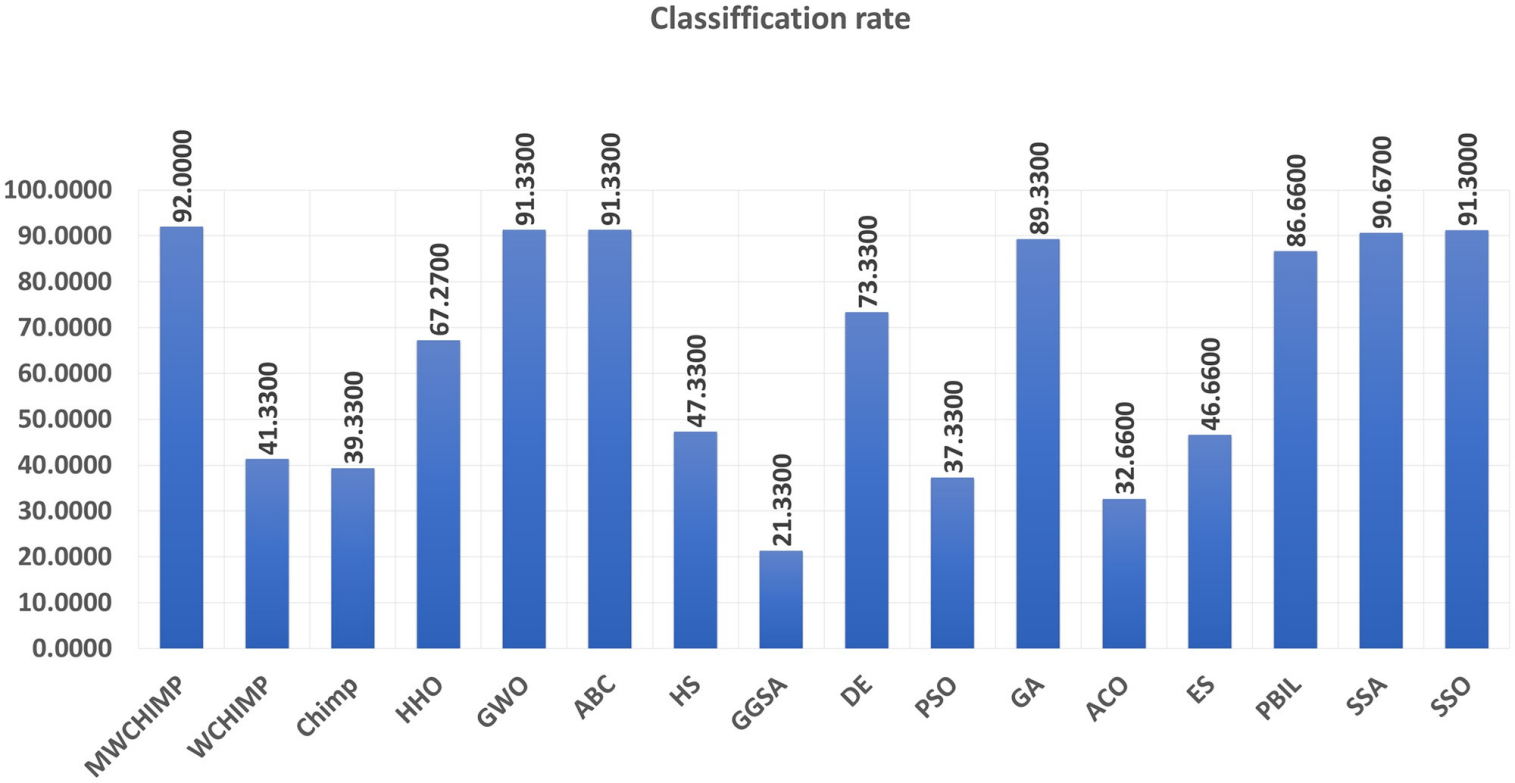
The classification rate of algorithms for IRIS.

**Fig 28:**
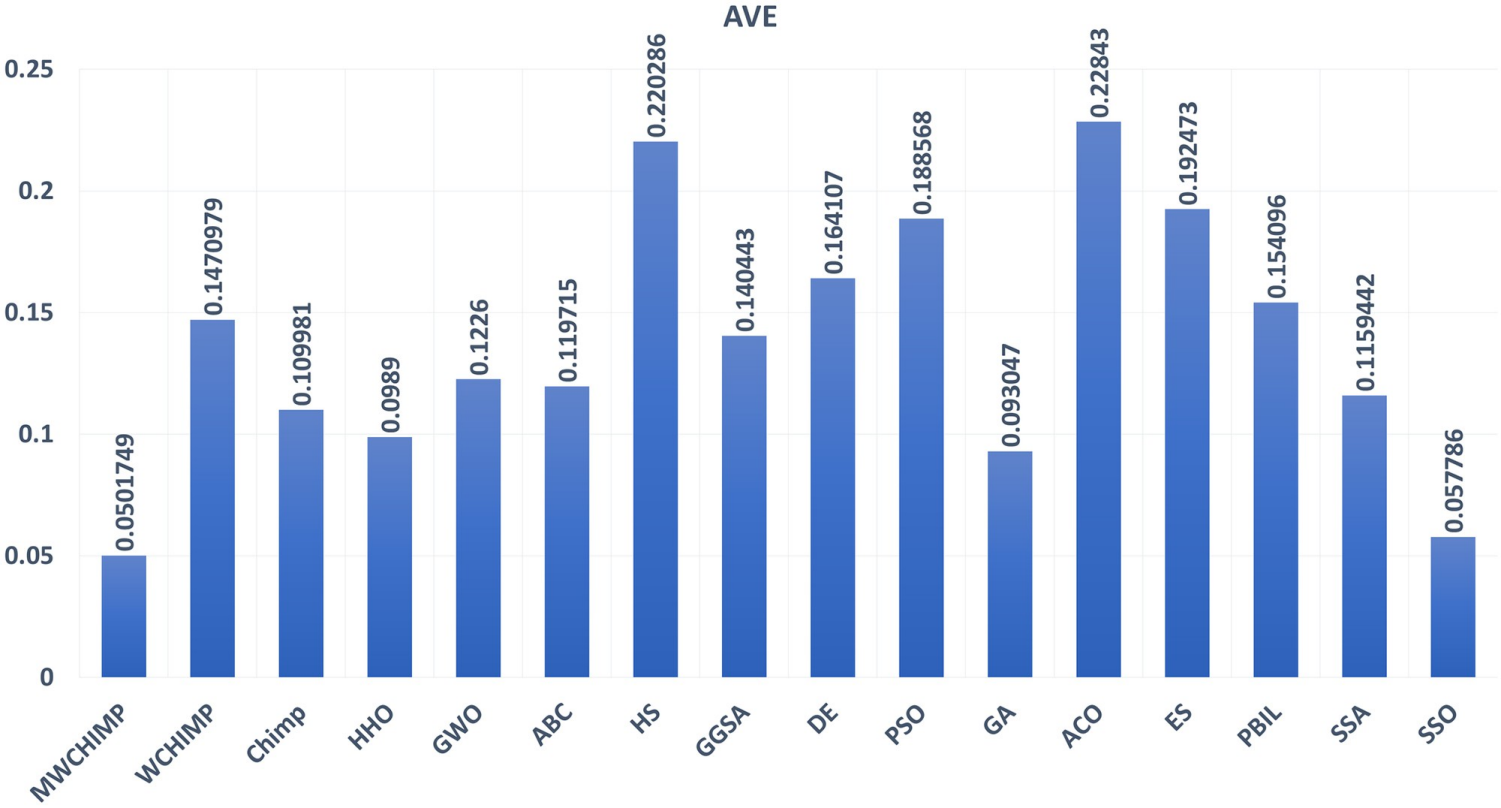
The AVE of algorithms for heart.

**Fig 29:**
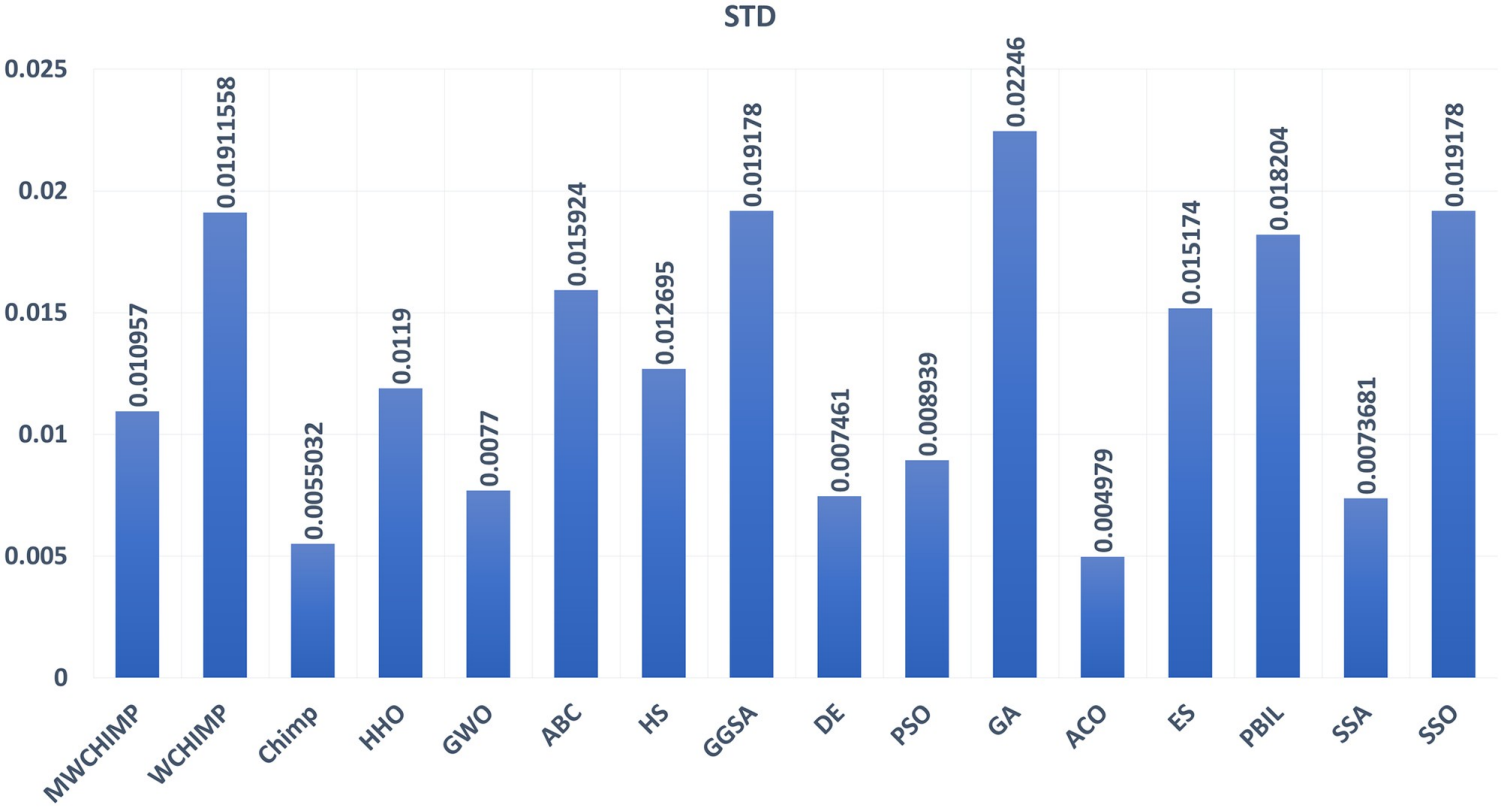
The STD of algorithms for heart.

**Fig 30:**
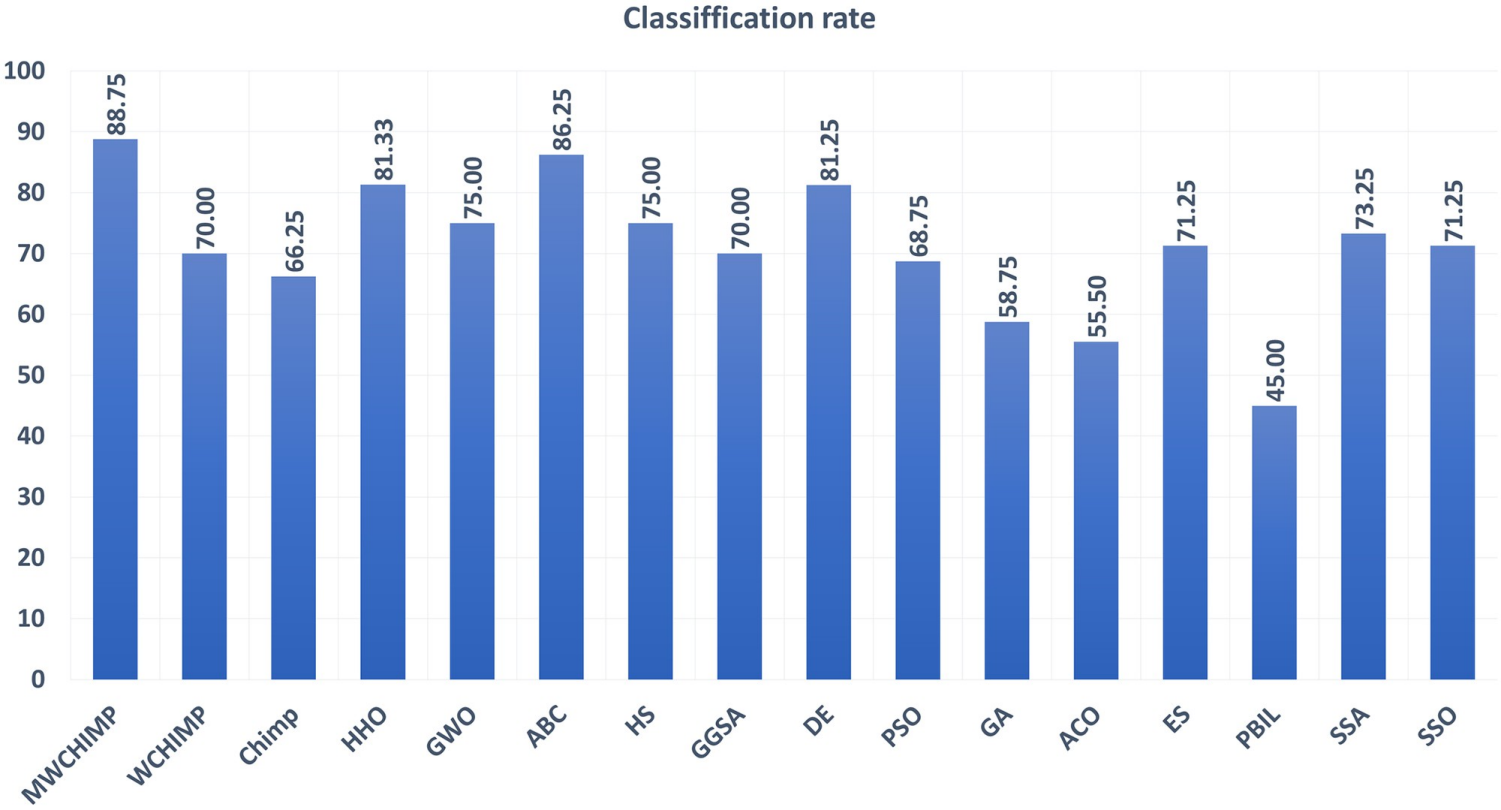
The classification rate of algorithms for heart.

**Table 8:** The XOR dataset’s statistical results.

Algorithm	AVE	STD	CR (%)
MWChOA	**3.67e^−06^**	**8.78e^−06^**	**100.00**
WChOA	0.030070	0.026822	87.50
ChOA	0.145703	0.066154	25
HHO	6.99*e*^−03^	1.37*e*^−02^	76.25
GWO	0.009410	0.029500	**100.00**
PSO	0.084050	0.035945	37.50
HS	0.095153	0.036786	62.50
GGSA	0.174183	0.025994	25.00
DE	0.038084	0.025033	87.50
GA	0.000181	0.000413	**100.00**
ACO	0.180328	0.025268	62.50
ES	0.118739	0.011574	62.50
PBIL	0.030228	0.039668	62.50
SSA	0.0001202	0.0003828	100.00
SSO	4.7098*e*^−05^	5.1662*e*^−05^	**100.00**

**Table 9:** The breast cancer dataset’s statistical results.

Algorithm	AVE	STD	CR (%)
WMCHOA	**0.0011**	8.62423*e*^−05^	**99.00**
WChOA	0.0012	0.0035	90.00
ChOA	0.01585	0.0035	88.00
HHO	0.00167	7.58*e*^−05^	98.50
GWO	0.0012	**7.4498e^−05^**	**99.00**
ABC	0.008903	0.001956	90.00
HS	0.034616	0.004431	79.00
GGSA	0.003676	0.001338	97.00
DE	0.029071	0.002742	78.00
PSO	0.034881	0.002472	11.00
GA	0.003026	0.001500	98.00
ACO	0.013510	0.002137	40.00
ES	0.040320	0.002470	06.00
PBIL	0.032009	0.003065	07.00
SSA	0.0063190	0.0049316	94.50
SSO	0.0016140	0.00019116	98.00

**Table 10:** The iris dataset’s statistical results.

Algorithm	AVE	STD	CR (%)
MWChOA	0.030421	0.004220	**92.00**
WChOA	0.155616	0.091370	41.3333
ChOA	0.2877	0.01611	39.33
HHO	0.0655	0.0988	67.27
GWO	0.0229	0.0032	91.333
ABC	0.029622	0.002205	91.333
HS	0.386776	0.064750	47.333
GGSA	0.182440	0.069217	21.333
DE	0.126699	0.026275	73.333
PSO	0.228680	0.057235	37.33
GA	0.089912	0.123638	89.33
ACO	0.405979	0.053775	32.66
ES	0.314340	0.052142	46.66
PBIL	0.116067	0.036355	86.66
SSA	0.0541041	0.0778262	90.67
SSO	**0.022413**	**0.002642**	91.30

**Table 11:** The heart dataset’s statistical results.

Algorithm	AVE	STD	CR (%)
MWChOA	**0.050174**	**0.010957**	**88.75**
WChOA	0.147097	0.019115	70
ChOA	0.109981	0.005503	66.25
HHO	0.0989	0.0119	81.33
GWO	0.122600	0.007700	75.00
ABC	0.119715	0.015924	86.25
HS	0.220286	0.012695	75.00
GGSA	0.140443	0.019178	70.00
DE	0.164107	0.007461	81.25
PSO	0.188568	0.008939	68.75
GA	0.093047	0.022460	58.75
ACO	0.228430	0.004979	00.00
ES	0.192473	0.015174	71.25
PBIL	0.154096	0.018204	45.00
SSA	0.1159442	0.007368	73.25
SSO	0.057786	0.019178	71.25

### 5.9 The Wilcoxon test

We conducted a statistical test experiment of the Wilcoxon test *p*-value in order to improve the performance evaluation of the optimization method [108]. A non-parametric test is run at a significance threshold of 5% to see if the MWChOA findings differ from the best outcomes of the other algorithms used in the statistical technique. Table 12 displays the *p* values for all algorithms. A *p*-value of less than 0.05 is typically regarded as being sufficient support for the null hypothesis. Table 12 above demonstrates that only the Iris dataset and the *p*-value of ABC are larger than 0.05, while all other values are less than 0.05, demonstrating the effectiveness of the proposed MWChOA.

**Table 12:** p-values from the Wilcoxon rank-sum test compared the MWChOA with the PSO, ABC, HHO, HS, GGSA, and DE across data sets.

MWChOA vs	PSO	ABC	HHO	HS	GGSA	DE
3-bits XOR	3.38*e*^−03^	2.21*e*^−03^	2.21*e*^−03^	2.21*e*^−03^	2.21*e*^−03^	2.21*e*^−03^
Balloon	8*e*^−05^	8*e*^−05^	8*e*^−05^	8*e*^−05^	8*e*^−05^	8*e*^−05^
Breast cancer	6.30*e*^−04^	1.8*e*^−03^	6.30*e*^−04^	6.34*e*^−03^	6.30*e*^−04^	6.34*e*^−04^
Iris	2.21*e*^−03^	**8.32*e*^−02^**	2.21*e*^−03^	2.21*e*^−03^	2.21*e*^−03^	2.21*e*^−03^
Heart	6.4*e*^−04^	6.4*e*^−04^	6.4*e*^−04^	6.4*e*^−04^	6.4*e*^−04^	6.3*e*^−04^

## 6 Conclusion and future works

Swarm intelligence algorithms (S) have been applied to solve many real-world problems. One of these problems is training the feed-forward neural network (FNN). However most of these algorithms suffer from slow convergence and are stuck in local optima. To overcome these issues, we propose a modified version of the standard Chimp Optimization algorithm (ChOA). The proposed algorithm is called a modified weighted chimp optimization algorithm (MWChOA). The proposed algorithm uses three leaders solution instead of four leaders in the standard ChOA and the weighted chimp optimization algorithm (WChOA). Reducing the number of the selected leaders’ solutions in the proposed MChOA improves the results and increases the accuracy of the obtained results. To test the efficiency of the proposed MWChOA, we test it on Eleven benchmark datasets and compared it against 16 SI algorithms. In future work, we will apply the proposed algorithm to train the most known deep learning algorithm such as Convolution neural networks (CNNs), and recurrent neural networks (RNNs) to solve many real-world applications.

## Supporting information

S1 Dataset (XLSX)

S2 Dataset (XLSX)

S3 Dataset (XLSX)

S4 Dataset (XLSX)

S5 Dataset (XLSX)

S6 Dataset (XLSX)

S7 Dataset (XLSX)

S8 Dataset (XLSX)

## References

[1] Zheng W., Xun Y., Wu X., Deng Z., Chen X., & Sui Y. A comparative study of class rebalancing methods for security bug report classification. IEEE Transactions on Reliability, 2021. 70(4), 1658–1670. doi: 10.1109/TR.2021.3118026

[2] Zheng W., Liu X., & Yin L. Research on image classification method based on improved multi-scale relational network. PeerJ Computer Science,2021. 7, e613. doi: 10.7717/peerj-cs.613 34395859 PMC8323718

[3] Zheng W., Yin L., Chen X., Ma Z., Liu S., & Yang B. Knowledge base graph embedding module design for Visual question answering model. Pattern Recognition, 2021.120, 108153. doi: 10.1016/j.patcog.2021.108153

[4] Ma Z., Zheng W., Chen X., & Yin L. Joint embedding VQA model based on dynamic word vector. PeerJ Computer Science,2021. 7, e353. doi: 10.7717/peerj-cs.353 33817003 PMC7959642

[5] Xu L., Liu X., Tong D., Liu Z., Yin L., & Zheng W. Forecasting Urban Land Use Change Based on Cellular Automata and the PLUS Model. Land,2022. 11(5), 652. doi: 10.3390/land11050652

[6] Lu S., Ban Y., Zhang X., Yang B., Yin L., Liu S., et al. Adaptive control of time delay teleoperation system with uncertain dynamics. Frontiers in Neurorobotics,2022. 152. doi: 10.3389/fnbot.2022.928863 35937561 PMC9354696

[7] Liu G. Data collection in mi-assisted wireless powered underground sensor networks: directions, recent advances, and challenges. IEEE Communications Magazine,2021. 59(4), 132–138. doi: 10.1109/MCOM.001.2000921

[8] Jia T., Cai C., Li X., Luo X., Zhang Y., & Yu X. Dynamical community detection and spatiotemporal analysis in multilayer spatial interaction networks using trajectory data. International Journal of Geographical Information Science,2022. 1–22.

[9] Liu X., Zhao J., Li J., Cao B., & Lv Z. Federated neural architecture search for medical data security. IEEE Transactions on Industrial Informatics, 2022.18(8), 5628–5636. doi: 10.1109/TII.2022.3144016

[10] Zhang Z., Luo C., & Zhao Z. Application of probabilistic method in maximum tsunami height prediction considering stochastic seabed topography. Natural Hazards,2020. 104(3), 2511–2530. doi: 10.1007/s11069-020-04283-3

[11] Hong T., Guo S., Jiang W., & Gong S. Highly Selective Frequency Selective Surface With Ultrawideband Rejection. IEEE Transactions on Antennas and Propagation, 2021. 70(5), 3459–3468. doi: 10.1109/TAP.2021.3137453

[12] Xu K. D., Weng X., Li J., Guo Y. J., Wu R., Cui J., et al. 60-GHz third-order on-chip bandpass filter using GaAs pHEMT technology. Semiconductor Science and Technology,2022. 37(5), 055004. doi: 10.1088/1361-6641/ac5bf8

[13] Li A., Masouros C., Swindlehurst A. L., & Yu W. 1-bit massive MIMO transmission: Embracing interference with symbol-level precoding. IEEE Communications Magazine,2021. 59(5), 121–127. doi: 10.1109/MCOM.001.2000601

[14] Wang Z., Ramamoorthy R., Xi X., & Namazi H. Synchronization of the neurons coupled with sequential developing electrical and chemical synapses. Mathematical Biosciences and Engineering, 2022.19(2), 1877–1890. doi: 10.3934/mbe.2022088 35135233

[15] Dai B., Zhang B., Niu Z., Feng Y., Liu Y., & Fan Y. A Novel Ultrawideband Branch Waveguide Coupler With Low Amplitude Imbalance. IEEE Transactions on Microwave Theory and Techniques,2022. 70(8), 3838–3846. doi: 10.1109/TMTT.2022.3186326

[16] NIU Z., ZHANG B., DAI B., ZHANG J., SHEN F., HU Y., et al. 220 GHz Multi Circuit Integrated Front End Based on Solid State Circuits for High Speed Communication System. Chinese Journal of Electronics,2022. 31(3), 569–580. doi: 10.1049/cje.2021.00.295

[17] Luo G., Yuan Q., Li J., Wang S., & Yang F. Artificial intelligence powered mobile networks: From cognition to decision. IEEE Network,2022. 36(3), 136–144. doi: 10.1109/MNET.013.2100087

[18] MANGASARIAN Olvi L.; STREET W. Nick; WOLBERG William H. Breast cancer diagnosis and prognosis via linear programming. Operations Research, 1995, 43.4: 570–577. doi: 10.1287/opre.43.4.570

[19] BEBIS George; GEORGIOPOULOS Michael. Feed-forward neural networks. IEEE Potentials, 1994, 13.4: 27–31. doi: 10.1109/45.329294

[20] Bishop C. Improving the generalization properties of radial basis function neural networks. Neural computation,1991. 3(4), 579–588. doi: 10.1162/neco.1991.3.4.579 31167338

[21] Ni, H., Yi, J., Wen, Z., & Tao, J. Recurrent neural network based language model adaptation for accent mandarin speech. In Chinese Conference on Pattern Recognition 2016.(pp. 607–617). Springer, Singapore.

[22] Ovtcharov K., Ruwase O., Kim J. Y., Fowers J., Strauss K., & Chung E. S. Accelerating deep convolutional neural networks using specialized hardware. Microsoft Research Whitepaper,2015. 2(11), 1–4.

[23] Fan C., Zhou Y., & Tang Z. Neighborhood centroid opposite-based learning Harris Hawks optimization for training neural networks. Evolutionary Intelligence, 2021. 14(4), 1847–1867. doi: 10.1007/s12065-020-00465-x

[24] Luo G., Zhang H., Yuan Q., Li J., & Wang F. Y. ESTNet: Embedded Spatial-Temporal Network for Modeling Traffic Flow Dynamics. IEEE Transactions on Intelligent Transportation Systems.2022.

[25] Sui T., Marelli D., Sun X., & Fu M. Multi-sensor state estimation over lossy channels using coded measurements. Automatica,2020. 111, 108561. doi: 10.1016/j.automatica.2019.108561

[26] Yang C.C., Prasher S.O., Landry J.A., DiTommaso A. Application of artificial neural networks in image recognition and classification of crop and weeds. Canadian agricultural engineering,2000. 42(3), 147–152.

[27] Adwan O., Faris H., Jaradat K., Harfoushi O., Ghatasheh N. Predicting customer churn in telecom industry using multilayer preceptron neural networks Modeling and analysis. Life Science Journal, 2014. 11(3), 75–81

[28] Leshno M., Lin V. Y., Pinkus A.,& Schocken S. Multilayer feedforward networks with a nonpolynomial activation function can approximate any function. Neural networks, 1993.6(6), 861–867. doi: 10.1016/S0893-6080(05)80131-5

[29] Amato F., López A., Peña-Méndez E. M., Vaňhara P., Hampl A., & Havel J. Artificial neural networks in medical diagnosis. Journal of applied biomedicine, 2013.11(2), 47–58. doi: 10.2478/v10136-012-0031-x

[30] Lo S. C. B., Chan H. P., Lin J. S., Li H., Freedman M. T., & Mun S. K. Artificial convolution neural network for medical image pattern recognition. Neural networks, 1995. 8(7-8), 1201–1214. doi: 10.1016/0893-6080(95)00061-5

[31] Golfinopoulos E., Tourville J. A., & Guenther F. H. The integration of large-scale neural network modeling and functional brain imaging in speech motor control. Neuroimage, 2010. 52(3), 862–874. doi: 10.1016/j.neuroimage.2009.10.023 19837177 PMC2891349

[32] Faris H., Alkasassbeh M., & Rodan A. Artificial Neural Networks for Surface Ozone Prediction: Models and Analysis. Polish Journal of Environmental Studies,2014. 23(2).

[33] Engelbrecht A.P. Supervised learning neural networks. Computational Intelligence: An Introduction, 2nd ed., 2007.pp. 27–54. Wiley, Singapore.

[34] Melin P., & Castillo O. Hybrid intelligent systems for pattern recognition using soft computing: an evolutionary approach for neural networks and fuzzy systems. Springer Science & Business Media,2005.(Vol. 172).

[35] Stanley, K.O. Efficient reinforcement learning through evolving neural network topologies. In Proceedings of the 4th Annual Conference on genetic and evolutionary computation 2002 (pp. 569–577)

[36] Sivagaminathan R.K., Ramakrishnan S. A hybrid approach for feature subset selection using neural networks and ant colony optimization. Expert systems with applications,2007. 33(1), 49–60 doi: 10.1016/j.eswa.2006.04.010

[37] Zhang N. An online gradient method with momentum for two layer feed forward neural networks. Applied Mathematics and Computation,2009. 212(2), 488–497 doi: 10.1016/j.amc.2009.02.038

[38] Cao B., Zhao J., Liu X., Arabas J., Tanveer M., Singh A. K., et al. Multiobjective evolution of the explainable fuzzy rough neural network with gene expression programming. IEEE Transactions on Fuzzy Systems.2022. doi: 10.1109/TFUZZ.2022.3141761

[39] Wang H., Gao Q., Li H., Wang H., Yan L., & Liu G. A Structural Evolution-Based Anomaly Detection Method for Generalized Evolving Social Networks. The Computer Journal,2022. 65(5), 1189–1199. doi: 10.1093/comjnl/bxaa168

[40] Merkl D., & Rauber A. Document classification with unsupervised artificial neural networks. In Soft computing in information retrieval.2000 (pp. 102–121). Physica, Heidelberg.

[41] Aljarah I., & Ludwig S. A. A scalable mapreduce-enabled glowworm swarm optimization approach for high dimensional multimodal functions. International Journal of Swarm Intelligence Research (IJSIR),2016. 7(1), 32–54. doi: 10.4018/IJSIR.2016010102

[42] Zhang Y., Liu F., Fang Z., Yuan B., Zhang G., & Lu J. Learning from a complementary-label source domain: theory and algorithms. IEEE Transactions on Neural Networks and Learning Systems. (2021). 34138722 10.1109/TNNLS.2021.3086093

[43] Alboaneen, D. A., Tianfield, H., & Zhang, Y. Glowworm swarm optimisation for training multi-layer perceptrons. In Proceedings of the Fourth IEEE/ACM International Conference on Big Data Computing, Applications and Technologies.2017. (pp. 131–138).

[44] Zhong L., Fang Z., Liu F., Yuan B., Zhang G., & Lu J. Bridging the theoretical bound and deep algorithms for open set domain adaptation. IEEE transactions on neural networks and learning systems.2021. doi: 10.1109/TNNLS.2021.3119965 34714753

[45] Seiffert U. Multiple layer perceptron training using genetic algorithms. In ESANN.2001 (pp. 159–164).

[46] Zhang J. R., Zhang J., Lok T. M., & Lyu M. R. A hybrid particle swarm optimization–back-propagation algorithm for feedforward neural network training. Applied mathematics and computation,2007. 185(2), 1026–1037.

[47] Wienholt, W. Minimizing the system error in feedforward neural networks with evolution strategy. In International Conference on Artificial Neural Networks.1993 (pp. 490–493). Springer, London.

[48] Mavrovouniotis M., & Yang S. Training neural networks with ant colony optimization algorithms for pattern classification. Soft Computing,2015. 19(6), 1511–1522. doi: 10.1007/s00500-014-1334-5

[49] Nawi N. M., Khan A., Rehman M. Z., Herawan T., & Deris M. M. Comparing performances of cuckoo search based neural networks. In Recent Advances on Soft Computing and Data Mining. 2014. (pp. 163–172). Springer, Cham.

[50] Brajevic, I., & Tuba, M. Training feed-forward neural networks using firefly algorithm. In Proceedings of the 12th International Conference on Artificial Intelligence, Knowledge Engineering and Data Bases (AIKED’13)2013. (pp. 156–161).

[51] Galić, E., & Höhfeld, M. Improving the generalization performance of multi-layer-perceptrons with population-based incremental learning. In International Conference on Parallel Problem Solving from Nature.1996 (pp. 740–750). Springer, Berlin, Heidelberg.

[52] Ilonen J., Kamarainen J. K., & Lampinen J. Differential evolution training algorithm for feed-forward neural networks. Neural Processing Letters,2003 17(1), 93–105. doi: 10.1023/A:1022995128597

[53] Karaboga, D., Akay, B., & Ozturk, C. Artificial bee colony (ABC) optimization algorithm for training feed-forward neural networks. In International conference on modeling decisions for artificial intelligence,2007. (pp. 318–329). Springer, Berlin, Heidelberg.

[54] Xi Y., Jiang W., Wei K., Hong T., Cheng T., & Gong S. Wideband RCS Reduction of Microstrip Antenna Array Using Coding Metasurface With Low Q Resonators and Fast Optimization Method. IEEE Antennas and Wireless Propagation Letters,2021. 21(4), 656–660.

[55] Li A., Spano D., Krivochiza J., Domouchtsidis S., Tsinos C. G., Masouros C., et al. A tutorial on interference exploitation via symbol-level precoding: overview, state-of-the-art and future directions. IEEE Communications Surveys & Tutorials,2020. 22(2), 796–839. doi: 10.1109/COMST.2020.2980570

[56] Boussaïd I. Lepagnot J., & Siarry P. A survey on optimization metaheuristics. Information sciences,2013. 237, 82–117. doi: 10.1016/j.ins.2013.02.041

[57] Li K., Thompson S., & Peng J. X. GA based neural network modeling of NOx emission in a coal-fired power generation plant. IFAC Proceedings Volumes,2002. 35(1), 281–286. doi: 10.3182/20020721-6-ES-1901.01198

[58] Sohn D., Mabu S., Shimada K., Hirasawa K., & Hu J. Training of multi-branch neural networks using RasID-GA. In 2007 IEEE Congress on Evolutionary Computation,2007. (pp. 2064–2070). IEEE.

[59] Zhang, C., Shao, H., & Li, Y. Particle swarm optimisation for evolving artificial neural network. In Smc 2000 conference proceedings. 2000 ieee international conference on systems, man and cybernetics.’cybernetics evolving to systems, humans, organizations, and their complex interactions’(cat. no. 0 (Vol. 4, pp. 2487–2490). IEEE.

[60] Garro B. A., & Vázquez R. A. Designing artificial neural networks using particle swarm optimization algorithms. Computational intelligence and neuroscience, 2015. doi: 10.1155/2015/369298 26221132 PMC4499655

[61] Mirjalili S., Hashim S. Z. M., & Sardroudi H. M. Training feedforward neural networks using hybrid particle swarm optimization and gravitational search algorithm. Applied Mathematics and Computation,2012. 218(22), 11125–11137. doi: 10.1016/j.amc.2012.04.069

[62] Socha K., & Blum C. An ant colony optimization algorithm for continuous optimization: application to feed-forward neural network training. Neural computing and applications,2007. 16(3), 235–247. doi: 10.1007/s00521-007-0084-z

[63] Blum, C., & Socha, K. Training feed-forward neural networks with ant colony optimization: An application to pattern classification. In Fifth International Conference on Hybrid Intelligent Systems (HIS’05),2005. (pp. 6-pp). IEEE

[64] Ozturk C., & Karaboga D. Hybrid artificial bee colony algorithm for neural network training. In 2011 IEEE congress of evolutionary computation (CEC),2011. (pp. 84–88). IEEE.

[65] Ghorbani M. A., Deo R. C., Karimi V., Kashani M. H.,& Ghorbani S. Design and implementation of a hybrid MLP-GSA model with multi-layer perceptron-gravitational search algorithm for monthly lake water level forecasting. Stochastic Environmental Research and Risk Assessment,2019. 33(1), 125–147. doi: 10.1007/s00477-018-1630-1

[66] Mirjalili, S., & Sadiq, A. S. Magnetic optimization algorithm for training multi layer perceptron. In 2011 IEEE 3rd international conference on communication software and networks,2011. (pp. 42–46). IEEE.

[67] Mirjalili S., Mirjalili S. M., & Lewis A. Grey wolf optimizer. Advances in engineering software,2014. 69, 46–61. doi: 10.1016/j.advengsoft.2013.12.007

[68] Pu X., Chen S., Yu X., & Zhang L. Developing a novel hybrid biogeography-based optimization algorithm for multilayer perceptron training under big data challenge. Scientific Programming, 2018. doi: 10.1155/2018/2943290

[69] Zhao R., Wang Y., Hu P., Jelodar H., Yuan C., Li Y., et al. Selfish herds optimization algorithm with orthogonal design and information update for training multi-layer perceptron neural network. Applied Intelligence,2019. 49(6), 2339–2381. doi: 10.1007/s10489-018-1373-1

[70] Xu J., & Yan F. Hybrid Nelder–Mead algorithm and dragonfly algorithm for function optimization and the training of a multilayer perceptron. Arabian Journal for Science and Engineering,2019. 44(4), 3473–3487. doi: 10.1007/s13369-018-3536-0

[71] Goerick, C., & Rodemann, T. Evolution strategies: an alternative to gradient-based learning. In Proceedings of the International Conference on Engineering Applications of Neural Networks,1996. (Vol. 1, pp. 479–482).

[72] Aljarah I., Faris H., Mirjalili S., Al-Madi N., Sheta A., & Mafarja M. Evolving neural networks using bird swarm algorithm for data classification and regression applications. Cluster Computing,2019. 22(4), 1317–1345. doi: 10.1007/s10586-019-02913-5

[73] Sağ T., & Abdullah Jalil Jalil Z. Vortex search optimization algorithm for training of feed-forward neural network. International Journal of Machine Learning and Cybernetics,2021. 12(5), 1517–1544. doi: 10.1007/s13042-020-01252-x

[74] Chatterjee R., Mukherjee R., Roy P. K., & Pradhan D. K. Chaotic oppositional-based whale optimization to train a feed forward neural network. Soft Computing,2022. 1–23.

[75] Gülcü Ş. Training of the feed forward artificial neural networks using dragonfly algorithm. Applied Soft Computing,2022. 109023. 10.1007/s00500-022-07592-w PMC9584244 36284902

[76] KUMAR B. S., & JAYARAJ D. ZEALOUS PARTICLE SWARM OPTIMIZATION BASED RELIABLE MULTI-LAYER PERCEPTRON NEURAL NETWORKS FOR AUTISM SPECTRUM DISORDER CLASSIFICATION. Journal of Theoretical and Applied Information Technology,2023. 101(1).

[77] Emambocus B. A. S., Jasser M. B., & Amphawan A. A Survey on the Optimization of Artificial Neural Networks Using Swarm Intelligence Algorithms. IEEE Access, 2023. 11, 1280–1294. doi: 10.1109/ACCESS.2022.3233596

[78] Khishe M., & Mosavi M. R. Classification of underwater acoustical dataset using neural network trained by Chimp Optimization Algorithm. Applied Acoustics,2020. 157, 107005. doi: 10.1016/j.apacoust.2019.107005

[79] Saffari A., Khishe M., & Zahiri S. H. Fuzzy-ChOA: an improved chimp optimization algorithm for marine mammal classification using artificial neural network. Analog Integrated Circuits and Signal Processing,2022. 111(3), 403–417. doi: 10.1007/s10470-022-02014-1 35291314 PMC8912427

[80] Cai C., Gou B., Khishe M., Mohammadi M., Rashidi S., Moradpour R., et al. Improved deep convolutional neural networks using chimp optimization algorithm for Covid19 diagnosis from the X-ray images. Expert Systems with Applications, 2023. 213, 119206. doi: 10.1016/j.eswa.2022.119206 36348736 PMC9633109

[81] Saffari A., Zahiri S. H., Khishe M., & Mosavi S. M. Design of a fuzzy model of control parameters of chimp algorithm optimization for automatic sonar targets recognition. Iranian journal of Marine technology,2022. 9(1), 1–14

[82] Mou J., Duan P., Gao L., Liu X., & Li J. An effective hybrid collaborative algorithm for energy-efficient distributed permutation flow-shop inverse scheduling. Future Generation Computer Systems,2022. 128, 521–537. doi: 10.1016/j.future.2021.10.003

[83] Gülcü Ş. An Improved Animal Migration Optimization Algorithm to Train the Feed-Forward Artificial Neural Networks. Arab J Sci Eng 47, 9557–9581, 2022. doi: 10.1007/s13369-021-06286-z 34777937 PMC8578534

[84] Bacanin N., Bezdan T., Zivkovic M., & Chhabra A. Weight optimization in artificial neural network training by improved monarch butterfly algorithm. In Mobile Computing and Sustainable Informatics (pp. 397–409).2022. Springer, Singapore.

[85] Bebis G, Georgiopoulos M. Feed-forward neural networks. Potentials IEEE,1994. 13:27–31. doi: 10.1109/45.329294

[86] Mirjalili S. How effective is the Grey Wolf optimizer in training multi-layer perceptrons. Applied Intelligence,2015. 43(1), 150–161. doi: 10.1007/s10489-014-0645-7

[87] Khishe M., & Mosavi M. R. Chimp optimization algorithm. Expert systems with applications,2020. 149, 113338. doi: 10.1016/j.eswa.2020.113338 PMC9633109 36348736

[88] Khishe M., Nezhadshahbodaghi M., Mosavi M. R., & Martín D. A weighted chimp optimization algorithm. IEEE Access, 2021. 9, 158508–158539.

[89] http://archive.ics.uci.edu/ml/

[90] Maymounkov P., & Mazieres D. Kademlia: A peer-to-peer information system based on the xor metric. In International Workshop on Peer-to-Peer Systems,2002. (pp. 53–65). Springer, Berlin, Heidelberg.

[91] Klenk K. F., Bhartia P. K., Hilsenrath E., & Fleig A. J. Standard ozone profiles from balloon and satellite data sets. Journal of Applied Meteorology and Climatology,1983. 22(12), 2012–2022. doi: 10.1175/1520-0450(1983)022<2012:SOPFBA>2.0.CO;2

[92] Prentice R. L., & Gloeckler L. A. Regression analysis of grouped survival data with application to breast cancer data. Biometrics,1978. 57–67. doi: 10.2307/2529588 630037

[93] Krzanowski W. J., & Lai Y. T. A criterion for determining the number of groups in a data set using sum-of-squares clustering. Biometrics,1988. 23–34. doi: 10.2307/2531893

[94] Abrahams J. P., Leslie A. G., Lutter R., & Walker J. E. Structure at 2.8 A resolution of F1-ATPase from bovine heart mitochondria. Nature,1994. 370 (6491), 621–628. doi: 10.1038/370621a0 8065448

[95] Cestnik, B., Kononenko, I., & Bratko, I. A knowledge-elicitation tool for sophisticated users. In Proceedings of the 2nd European Conference on European Working Session on Learning EWSL, (1987), (Vol. 87).

[96] Haberman, S. J. Generalized residuals for log-linear models proceedings of the 9th International Biometrics Conference, (1976).

[97] McDermott, J., & Forsyth, R. S. Diagnosing a disorder in a classification benchmark. Pattern Recognition Letters, 73, (2016), 41–43.

[98] Sigillito V. G., Wing S. P., Hutton L. V., & Baker K. B. Classification of radar returns from the ionosphere using neural networks. Johns Hopkins APL Technical Digest, 10(3), (1989), 262–266.

[99] Hong Z. Q., & Yang J. Y. Optimal discriminant plane for a small number of samples and design method of classifier on the plane. pattern recognition, 24(4), (1991), 317–324. doi: 10.1016/0031-3203(91)90074-F

[100] Smith J. W., Everhart J. E., Dickson W. C., Knowler W. C., & Johannes R. S. Using the ADAP learning algorithm to forecast the onset of diabetes mellitus. In Proceedings of the annual symposium on computer application in medical care, (1988), (p. 261). American Medical Informatics Association.

[101] Tharwat A. Classification assessment methods. Applied Computing and Informatics (2020).

[102] Karaboga D., & Basturk B. A powerful and efficient algorithm for numerical function optimization: artificial bee colony (ABC) algorithm. Journal of global optimization,2007. 39(3), 459–471. doi: 10.1007/s10898-007-9149-x

[103] Geem Z. W., Kim J. H., & Loganathan G. V. A new heuristic optimization algorithm: harmony search. simulation,2001. 76(2), 60–68. doi: 10.1177/003754970107600201

[104] Mirjalili S., & Lewis A. Adaptive gbest-guided gravitational search algorithm. Neural Computing and Applications, 2014. 25(7), 1569–1584. doi: 10.1007/s00521-014-1640-y

[105] Liu Y. P., Wu M. G., & Qian J. X. Evolving neural networks using the hybrid of ant colony optimization and BP algorithms. In International Symposium on Neural Networks, 2006. (pp. 714–722). Springer, Berlin, Heidelberg.

[106] Bairathi D., & Gopalani D. Numerical optimization and feed-forward neural networks training using an improved optimization algorithm: multiple leader salp swarm algorithm. Evolutionary Intelligence,2021. 14(3), 1233–1249. doi: 10.1007/s12065-019-00269-8

[107] Pereira L. A., Rodrigues D., Ribeiro P. B., Papa J. P., & Weber S. A. Social-spider optimization-based artificial neural networks training and its applications for Parkinson’s disease identification. In 2014 IEEE 27th international symposium on computer-based medical systems, 2014.(pp. 14–17). Ieee.

[108] Yang X. S. Engineering optimization: an introduction with metaheuristic applications. John Wiley & Sons.

